# Myosin F controls actin organization and dynamics in *Toxoplasma gondii*

**DOI:** 10.1091/mbc.E23-12-0510

**Published:** 2024-03-12

**Authors:** Jacob A. Kellermeier, Aoife T. Heaslip

**Affiliations:** Department of Molecular and Cell Biology, University of Connecticut, Storrs, CT 06269; Université de Genève

## Abstract

Intracellular cargo transport is a ubiquitous cellular process in all eukaryotes. In many cell types, membrane bound cargo is associated with molecular motors which transport cargo along microtubule and actin tracks. In *Toxoplasma gondii* (*T. gondii*), an obligate intracellular parasite in the phylum Apicomplexa, organization of the endomembrane pathway depends on actin and an unconventional myosin motor, myosin F (MyoF). Loss of MyoF and actin disrupts vesicle transport, organelle positioning, and division of the apicoplast, a nonphotosynthetic plastid organelle. How this actomyosin system contributes to these cellular functions is still unclear. Using live-cell imaging, we observed that MyoF-EmeraldFP (MyoF-EmFP) displayed a dynamic and filamentous-like organization in the parasite cytosol, reminiscent of cytosolic actin filament dynamics. MyoF was not associated with the Golgi, apicoplast or dense granule surfaces, suggesting that it does not function using the canonical cargo transport mechanism. Instead, we found that loss of MyoF resulted in a dramatic rearrangement of the actin cytoskeleton in interphase parasites accompanied by significantly reduced actin dynamics. However, actin organization during parasite replication and motility was unaffected by the loss of MyoF. These findings revealed that MyoF is an actin organizing protein in *Toxoplasma* and facilitates cargo movement using an unconventional transport mechanism.

## Abstract (executive summary)

Myosin F (MyoF) is an apicomplexan specific myosin motor that is involved in vesicle transport, organelle positioning, and inheritance in *Toxoplasma gondii*. However, the mechanisms by which MyoF executes these essential processes are unknown.

We imaged actin organization in live parasites and show that cytosolic actin is highly dynamic and undergoes continual rearrangement. In the absence of Myosin F, actin is less dynamic and accumulates adjacent to the Golgi.

Thus, this paper identifies MyoF as a novel actin organizing protein in *T. gondii*.

## INTRODUCTION

Intracellular cargo transport is a conserved cellular process that is vital for normal cellular functions such as organelle positioning, communication, secretion, and uptake of molecules from the cell’s extracellular environment. The mechanisms of cargo transport have been intensively studied in mammalian cells and yeast (Hirokawa *et al.*, 2009; Hammer and Sellers, 2011; Reck-Peterson *et al.*, 2018), however mechanisms of cargo transport in other eukaryotic groups are poorly understood. In *Toxoplasma gondii (T. gondii)*, and other parasites in the Apicomplexan phylum, our understanding of cargo trafficking mechanisms is limited, and yet, parasite survival and pathogenicity critically depend on this cellular process.

Because *T. gondii* is an obligate intracellular parasite, survival depends on repeated “lytic” cycles of host cell invasion, replication, and egress. These processes in turn involve secretion of proteins from three types of secretory vesicles: the micronemes, rhoptries, and dense granules. Invasion is dependent on micronemes and rhoptries; sequential secretion of their proteomic contents enable parasites to attach and invade the host cell (Bisio and Soldati-Favre, 2019; Cova *et al.*, 2022). During invasion, the parasite hijacks a portion of the host cell membrane to form the parasitophorous vacuole (PV), where the parasite resides during the intracellular portion of its life cycle (Suss-Toby *et al.*, 1996; Pavlou *et al.*, 2018). Proteins secreted from dense granules are required for maturation of the PV and regulation of host gene expression and immune response pathways (Griffith *et al.*, 2022). Microneme proteins are also secreted at the end of the replication cycle to permeabilize the host cell and PV, and initiate motility (Kafsack *et al.*, 2009). Thus, the accurate trafficking of secretory proteins from their synthesis in the endoplasmic reticulum (ER) to the secretory organelles is essential for parasite survival.

After synthesis in the ER, secretory proteins are first trafficked apically towards the Golgi. Proteins destined for secretion move through the polarized endomembrane pathway, where microneme and rhoptry-destined proteins move to post-Golgi compartments for sorting and proteolytic maturation before finally being delivered to their respective secretory organelles (Parussini *et al.*, 2010; Kremer *et al.*, 2013; Venugopal and Marion, 2018), while dense granules form from post-Golgi vesicles (Griffith *et al.*, 2022). Despite its importance, the mechanisms that regulate organelle positioning and vesicle transport are not well understood.

We recently identified two components that are essential for the organization of the endomembrane pathway: actin and the myosin motor myosin F, (MyoF). These proteins are required for directed movement of vesicles including dense granules, and Rab11a and Rab6 vesicles (Heaslip *et al.*, 2016; Venugopal *et al.*, 2020; Carmeille *et al.*, 2021). Disruption of this actomyosin system also effects Golgi integrity, positioning of the post-Golgi compartments, and inheritance of a nonphotosynthetic plastid organelle called the apicoplast (Jacot *et al.*, 2013; Carmeille *et al.*, 2021; Devarakonda *et al.*, 2023). Even though the biological roles of MyoF and actin are well defined, mechanistic insight into how this molecular motor controls the movement and positioning of such a wide array of cargos is still lacking.

The mechanisms underlying intracellular cargo movement have been extensively studied in other species. The “canonical” cargo transport mechanism involves molecular motors which associate with cargo via their C-terminal tail domain and move cargo throughout the cell along cytoskeletal tracks. In metazoans, this long-distance transport is driven predominately by kinesin- and dynein-molecular motors moving on microtubules that are nucleated at a centrally positioned centrosome and radiate outwards to the plasma membrane (Reck-Peterson *et al.*, 2018). In budding and fission yeast, transport is driven by myosin motors, such as myosin V, moving on actin filament bundles (Hill *et al.*, 1996; Pruyne *et al.*, 1998). However, other cargo transport mechanisms have also been described such as bulk cargo movement in oocytes (Schuh, 2011). In this case, actin nucleators and myosin V bind the vesicle surface, with the nucleators forming an actin meshwork, while myosin V moves processively along these F-actin tracks to drive collective cargo transport. In another example, plant myosin XI drives endoplasmic vesicle movement along polarized actin filaments at the cell periphery causing cytoplasmic streaming which passively carries large organelles such as mitochondria, peroxisomes, and the Golgi (Tominaga and Ito, 2015). Thus, the mechanism of cargo transport depends on the organization of, and interactions between, membrane bound cargo, molecular motors, and their cytoskeletal tracks.

Elucidation of the cargo transport mechanism employed by MyoF and actin requires an understanding of the organization and dynamics of these components in relation to endomembrane organelles. However, visualization of actin within *T. gondii* is challenging, as conventional probes for labeling actin, such as phalloidin, do not bind to *T. gondii* actin (Dobrowolski *et al.*, 1997; Shaw and Tilney, 1999). Recently, visualization of F-actin dynamics in live parasites was made possible with the use of an Actin chromobody (ActinCB) which has revealed the existence of an extensive F-actin network in intracellular parasites (Periz *et al.*, 2017). In addition to a cytoplasmic network, actin is also contained within tubules that link parasites in the PV during replication and surrounds daughter parasites during their construction (Periz *et al.*, 2017). While *T. gondii* contains an extensive actin cytoskeleton, it remains unclear how actin and MyoF coordinate intracellular cargo transport. Thus, the goal of this study is to investigate how this actomyosin system facilitates this essential cellular process. Using live-cell microscopy, we demonstrate that MyoF and F-actin both have filamentous-like, dynamic organization in the parasite cytosol and MyoF does not appear to enrich on the surface of membrane-bound cargo. Loss of MyoF leads to a dramatic rearrangement of actin in the cytosol, revealing that MyoF functions to facilitate actin dynamics and organization to drive endomembrane positioning and movement.

## RESULTS

### MyoF and F-actin are dynamic within the cytosol

**FIGURE 1:**
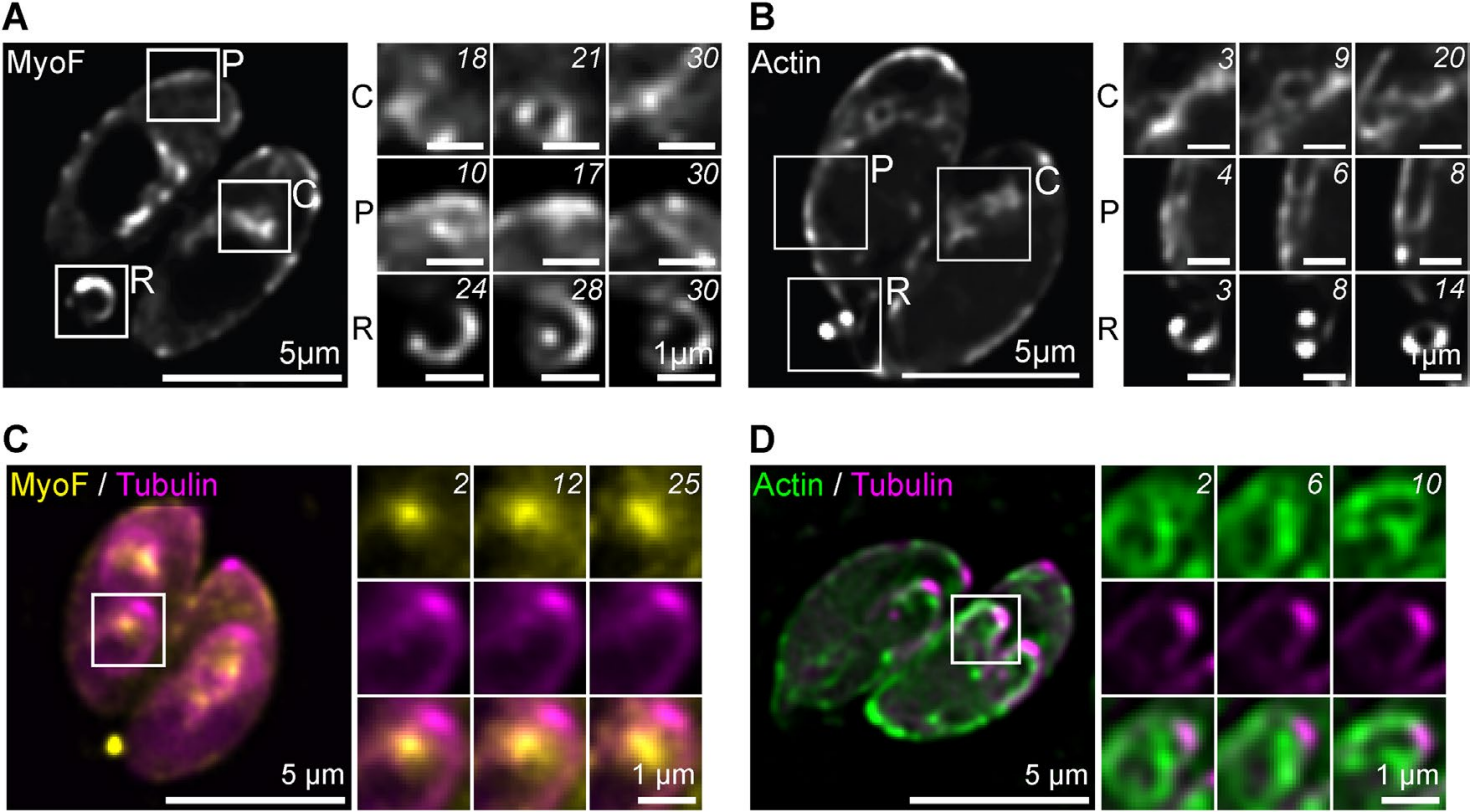
Actin and MyoF have dynamic filamentous-like localizations in the cytosol. Representative live images of (A) MyoF-EmFP or (B) ActinCB tagged with EmeraldGFP (ActinCB-EmFP) expressing parasites. Both proteins had cytosolic (C), peripheral (P), and RB (R) localizations. Imaging speed was 400 ms for MyoF-EmFP and 200 ms for ActinCB-EmFP parasites. White box indicates areas used to make insets which show the changes in the protein localizations over time. Time points in seconds are indicated in white italics. (C) MyoF-EmFP (yellow) and (D) ActinCB-EmFP (green) parasites transiently expressing mCherry-TubulinA1 (magenta), a marker of periphery of mother and daughter parasites, were imaged using live cell microscopy. During daughter cell construction, MyoF is localized apical to the nucleus and actin is localized at the periphery of the daughter parasites. Insets show protein localization at the indicated time for a cytosolic region of the cell (white box). Time points are indicated in white italics.

To understand the mechanism by which MyoF and F-actin regulate vesicle transport and organelle positioning, we visualized the organization and dynamics of these proteins using live-cell imaging. For visualization of MyoF, we used a previously generated parasite line where the endogenous MyoF gene was tagged at the C-terminus with EmeraldFP (MyoF-EmFP; Heaslip *et al.*, 2016). Consistent with previous results in fixed parasites (Jacot *et al.*, 2013; Heaslip *et al.*, 2016), MyoF had a predominately cytosolic localization with enrichment at the apical end of the parasite adjacent to the nucleus. The observed cytosolic signal of MyoF was highly dynamic and filamentous-like, with extensions from the apinuclear region to the apical tips of the parasite (Figure 1A, cytosol; Supplemental Video S1). MyoF was also enriched at the cell periphery in all parasites observed (Figure 1A; periphery; Supplemental Video S1). In 73 ± 24% of parasites, we also observed MyoF signal within the residual body (RB), where it was predominately localized at the RB perimeter (Figure 1A, RB; Supplemental Figure S1A).

**Figure:** Movie S1 *Top row*. MyoF‐EmFP (*left*) and ActinCB‐EmFP (*right*) organization during interphase Insets indicate cytosolic, peripheral, and residual body signal. Imaging speeds were 2.5 and 4.9 fps, respectively. Playback is 3x and 6x real time. *Bottom row*. Bottom panels show MyoF‐EmFP (yellow; left) and MyoF‐mAID:ActinCB‐EmFP (Green; right) parasites transiently expressing pTub‐mCherry‐TubulinA1 (magenta) to mark the tubulin cytoskeleton of daughter parasites. Insets show MyoF and actin localized to the growing daughter cells. Imaging speeds were 1 and 1.5 fps, respectively. Playback is 7.5x and 4.85x real time.

For visualization of F-actin, a single copy of the actin-chromobody fused to EmFP (hereafter, referred to as ActinCB) was expressed under the control of the minimal DHFR promoter from the dispensable *Ku80* locus in a parental line where MyoF was tagged with the auxin inducible degron (Supplemental Figure S2, A and C; Carmeille *et al.*, 2021). Transient expression of the chromobody at high levels disrupts actin dependent processes (Periz *et al.*, 2017). Therefore, we performed a plaque assay to determine whether stable expression of the chromobody affected the parasite’s lytic cycle. Comparison of the chromobody expressing parasites with the parental line revealed plaque formation for both lines (Supplemental Figure S1B). In addition, the ActinCB expressing parasites have been continually cultured for hundreds of passages without loss of ActinCB expression. Filamentous-like actin structures were enriched at the apical end of the parasite cytosol and were highly dynamic, undergoing continual rearrangement (Figure 1B; Supplemental Video S1). Similar to what was observed for MyoF, actin was also enriched at the parasite periphery and found in the RB in 83 ± 9.3% of parasites (Figure 1B; Supplemental Figure S1A). Due to the resolution limit of light microscopy, we are unable to determine whether these filamentous-like structures are individual actin filaments or bundles, nor are we able to discern actin filament lengths. Thus, reference to the filament or bundle organization of MyoF and actin are to provide a qualitative description of protein organization.

To determine whether actin structures could be observed in fixed cells, we tested a number of fixation conditions and compared these to the organization of actin in live samples. In all fixation conditions tested, parasite actin signal appeared diffuse in comparison to the filamentous organization obtained from live cells (Supplemental Figure S3). While the chromobody signal within RB was still visible, the diffuse cytosolic signal prevented actin imaging in fixed cells, a necessary step for obtaining higher resolution images of the cytosolic actin network.

Next, to determine how the localization and dynamics of MyoF and actin changed during cell division, we transiently expressed mCherry-tagged TubulinA1 to visualize the subpellicular microtubule array of both mother and daughter cells during division. During daughter cell formation, we observed MyoF localized to the central region of the nascent daughter cells, reminiscent of its localization during interphase close to the nucleus (Figure 1C; Supplemental Video S1). In contrast, dynamic F-actin was found within the daughter cell cytosol as well as with the periphery of daughter parasites, consistent with previous work (Periz *et al.*, 2017; Figure 1D; Supplemental Video S1).

### MyoF and F-actin localize apical of the Golgi

To investigate the spatial relationship between MyoF, actin, and membranous cargo, we colocalized MyoF and actin in relation to the Golgi, apicoplast, and dense granules. We chose to visualize these organelles because their transport and/or localization require both MyoF and actin (Jacot *et al.*, 2013; Heaslip *et al.*, 2016; Carmeille *et al.*, 2021).

**FIGURE 2:**
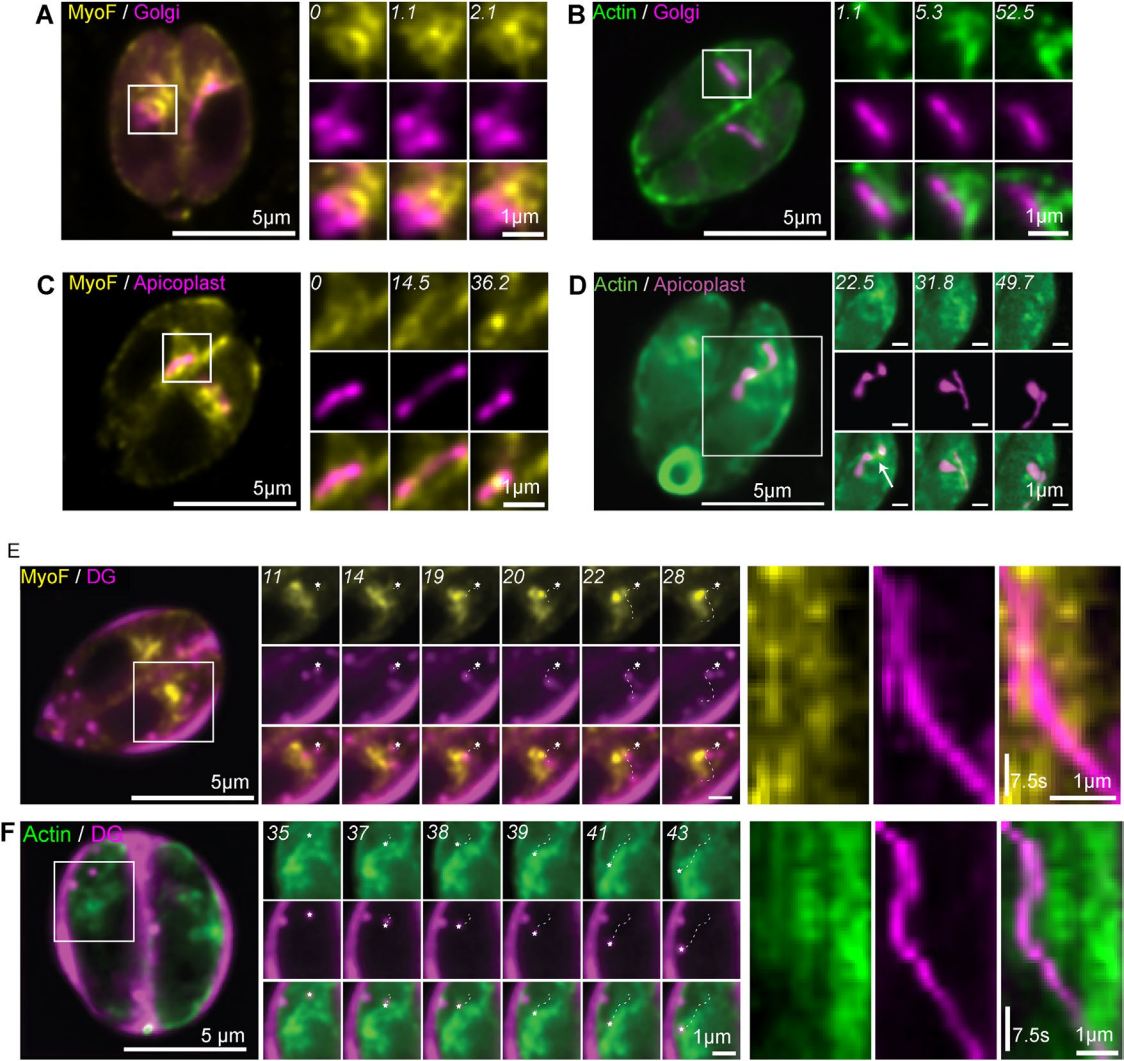
MyoF and actin dynamically surround organelles on the endomembrane pathway. Representative images of (A) MyoF-EmFP (yellow) and (B) ActinCB-EmFP (green) parasites transiently expressing Grasp-mCherryFP (magenta), a marker of the Golgi. Parasites were imaged using epifluorescence live cell microscopy. White box indicates areas used to make insets which show MyoF and actin filament-like structures localized apical to the cis-Golgi. Time points are indicated in white italics. Representative images of (C) MyoF-EmFP (yellow) and (D) ActinCB-EmFP (green) parasites transiently expressing FNR-RFP (magenta), a marker of the apicoplast. Parasites were imaged using epifluorescence live cell microscopy during the elongation phase of apicoplast division. White box indicates areas used to make insets. Time points are indicated in white italics. During apicoplast branch formation (D), actin is enriched at the site of branch formation (white arrow) and then actin filaments surround the branch during elongation. Representative images of (E) MyoF-EmFP (yellow) and (F) ActinCB-EmFP (green) parasites transiently expressing SAG1ΔGPI-mCherry (magenta), a marker for the dense granules. Parasites were imaged using epifluorescence live cell microscopy. White box indicates areas used to make insets (middle) which show MyoF and actin filament-like structures dynamically surrounding dense granules. MyoF is not enriched on the vesicle surface. Time points are indicated in white italics. Dashed line indicates the region of interest (ROI) used to make the kymographs (right).

Because loss of MyoF and actin result in Golgi fragmentation (Carmeille *et al.*, 2021), we transiently expressed GRASP55-mCherry, a marker of the cis-Golgi (Pelletier *et al.*, 2002), in MyoF-EmFP and ActinCB-EmFP parasites and performed two-color fluorescence microscopy on live parasites. Both MyoF and actin localized apical of the Golgi (Figure 2, A and B). This is consistent with previous reports that cytosolic actin in interphase parasites is nucleated by Golgi-adjacent formin-2 (Figure 2B, cytosol; Supplemental Video S2; Tosetti *et al.*, 2019), though actin and MyoF signals did not overlap with the signal of GRASP55-mCherry (Figure 2, A and B; Supplemental Video S2).

**Figure:** Movie S2 MyoF‐EmFP (yellow) and MyoF‐mAID:ActinCB‐EmFP (green) parasites transiently expressing pTub‐GRASP‐mCherry (top row; magenta), pTub‐FNR‐RFP (middle row; magenta), or pTub‐SAG1ΔGPI‐mCherrry (bottom row; magenta). Top show shows MyoF and actin closely associated with the apical face of the Golgi. Imaging speed was 1 fps. Playback is 15.7x real time. Middle panels show MyoF and actin dynamically localized along the apicoplast during elongation. Imaging speed was 1.7 and 1.5 fps, respectively. Playback is 9x and 9.9x real time. Bottom panels show dynamics of MyoF and actin during directed dense granule motion events. Imaging speed was 1.3 and 1.8 fps, respectively. Playback is 5.65 x and 4.15x real time.

### MyoF and F-actin dynamically surround the apicoplast during apicoplast elongation

Immunofluorescence assays on fixed samples demonstrated that MyoF is enriched near the apicoplast during apicoplast division (Jacot *et al.*, 2013; Stortz *et al.*, 2019). Both MyoF and actin are essential for apicoplast dynamics during the elongation phase of apicoplast division where the organelle transitions between linear, branched, and U-shaped structures (Devarakonda *et al.*, 2023). Loss of these proteins results in apicoplast inheritance defects (Jacot *et al.*, 2013; Devarakonda *et al.*, 2023). We transiently expressed the apicoplast lumen marker FNR-RFP (Striepen *et al.*, 2000) in MyoF-EmFP and ActinCB-EmFP parasites and performed two-color fluorescence microscopy during the elongation phase of apicoplast division. Both MyoF and actin exhibited filamentous-like structures surrounding the elongating apicoplast and transiently interacted with its surface (Figure 2, C and D; Supplemental Video S2). This was particularly apparent during apicoplast branching. Actin accumulated at the point of branch formation and then dynamically surrounded the branch during extension (Figure 2D, white arrow). It is notable that MyoF was not stably associated with the apicoplast surface.

### MyoF is not enriched on the surface of dense granules

Transport of the dense granules and Rab vesicles is dependent on MyoF and actin (Heaslip *et al.*, 2016; Carmeille *et al.*, 2021). In other cell types, ensembles of cargo-transporting myosin motors, such as MyoVa, associate with vesicles using their globular tail domain and move processively on actin filaments to transport vesicles throughout the cell (Hammer and Sellers, 2011). To understand whether MyoF functions similarly to cargo transporters such as MyoVa, we investigated whether MyoF was enriched at the surface of dense granules. We transiently expressed a construct which directs fluorescent proteins to the dense granules, SAG1ΔGPI-mCherry (Striepen *et al.*, 1998; Heaslip *et al.*, 2016) in MyoF-EmFP and ActinCB-EmFP parasites to image dense granule movement in relation to the actomyosin cytoskeleton with 750 and 550 ms time resolution, respectively. As described previously (Heaslip *et al.*, 2016), dense granules exhibited stationary, diffusive, and directed movements in parasites, often switching between these modes of motion. Regardless of the mode of movement exhibited by dense granules, we never observed MyoF enrichment onto the surface of dense granules. Instead, filamentous and dynamic MyoF signal transiently surrounded the dense granules (Figure 2E; Supplemental Video S2). When we imaged dense granule transport in relation to actin, we observed dynamic masses of actin that moved in a concerted manner with the translocating dense granule (Figure 2F; Supplemental Video S2).

Collectively, this data indicates that MyoF and F-actin exhibit filament-like organizations that dynamically surround the compartments and vesicles in the endomembrane network, and that MyoF is not stably associated with the membranes of the Golgi, apicoplast, or dense granules. Thus, it appears that MyoF does not facilitate cargo transport and positioning using the same mechanism as other cargo-transporting myosins such as MyoVa.

### MyoF knockdown alters F-actin dynamics and organization in interphase parasites

**FIGURE 3:**
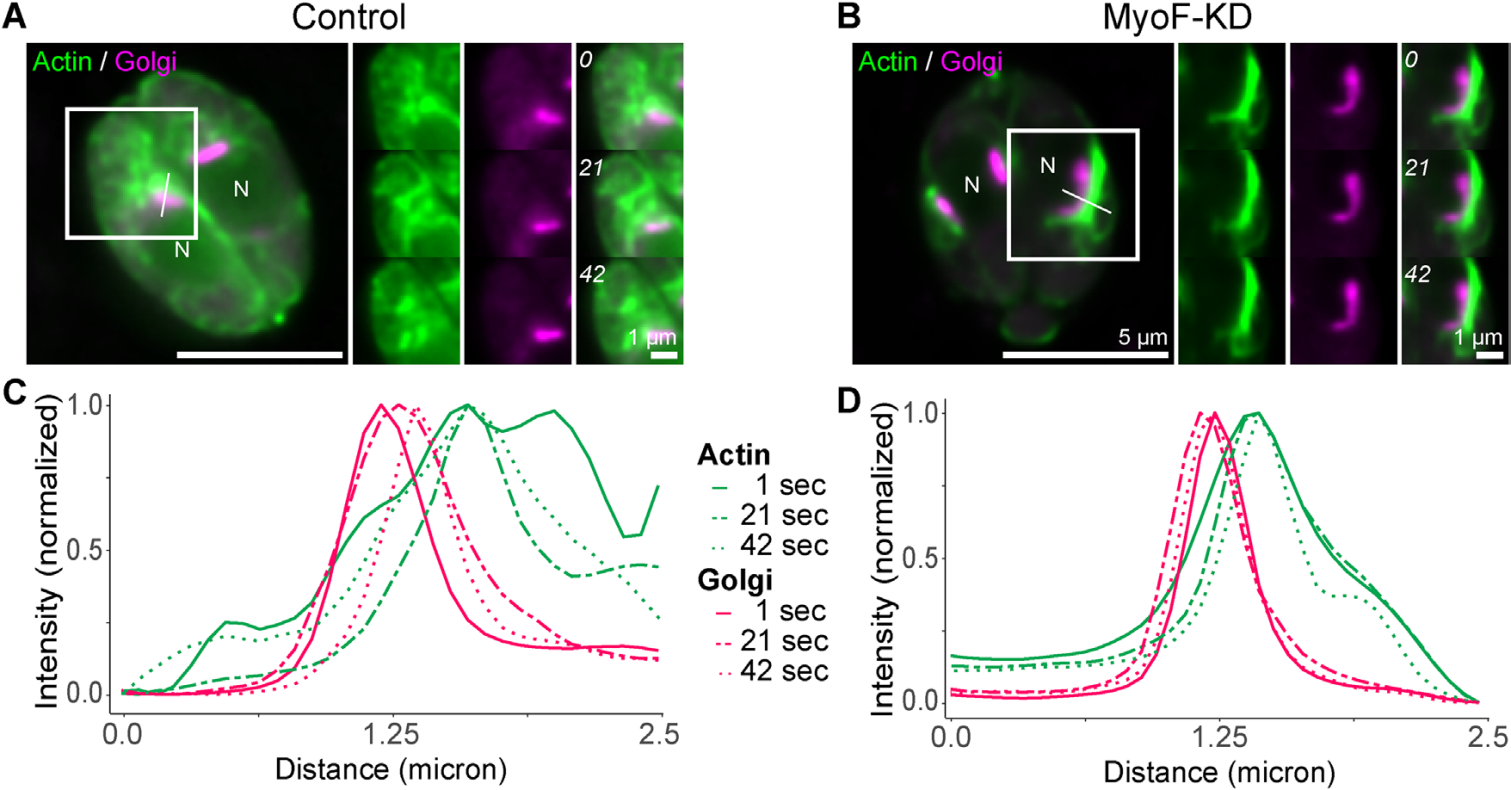
MyoF knockdown results in actin accumulation adjacent to the Golgi. Representative images of MyoF-mAID:ActinCB-EmFP parasites treated with vehicle control (EtOH) (A) or IAA for 15 h (B). Actin organization (green) was imaged in live parasites transiently expressing Grasp-mCherry, a marker for the Golgi (magenta). White lines indicate ROI used to make line scans. (C and D) Line scans show actin (green) or Golgi (magenta) distribution along the white line in (A) and (B) over time. Loss of MyoF results in accumulation of actin masses adjacent to the Golgi. N indicates the parasite nucleus.

The tail domain of MyoF has a predicted WD40 domain (Heaslip *et al.*, 2016), a common protein domain that is found on a number of actin binding proteins such as coronin (Salamun *et al.*, 2014; Olshina *et al.*, 2015). This observation led us to hypothesize that the WD40 domain of MyoF may interact with actin, allowing MyoF to remodel the actin cytoskeleton by dynamically crosslinking filaments. Thus, to test whether MyoF controls actin organization, we conditionally depleted MyoF in the MyoF-mAID:ActinCB-EmFP line by treating parasites with 3-indoleacetic acid (IAA) for 15–18 h before live-cell imaging of actin. To provide a reference point for any observable changes in actin organization or localization, we transiently expressed the Golgi marker GRASP55-mCherry. In control parasites, we observed a single Golgi at the apical end of the nucleus and the dynamic filamentous localization of actin as described above (Figure 3A; Supplemental Video S3). Line scans indicate the change in actin organization overtime (Figure 3C). Upon MyoF knockdown, the Golgi was fragmented and was associated with the apical, basal, and lateral sides of the nucleus as previously observed (Carmeille *et al.*, 2021). In the absence of MyoF, large masses of actin formed adjacent to each Golgi fragment, indicating that the loss of MyoF alters actin organization (Figure 3B; Supplemental Video S3). Line scans indicate that the organization of actin in MyoF knockdown parasites appeared less dynamic than in controls (Figure 3D).

**Figure:** Movie S3 MyoF‐mAID:ActinCB‐EmFP parasites transiently expressing GRASP‐mCherry in control and MyoF‐KD parasites. Top panels show actin dynamically associated with the apical face of the Golgi. Bottom panels show large masses of actin tightly associated with the Golgi. White box indicates area used to make inset panels. Imaging speed was 1 fps. Playback is 7.85x real time.

**FIGURE 4:**
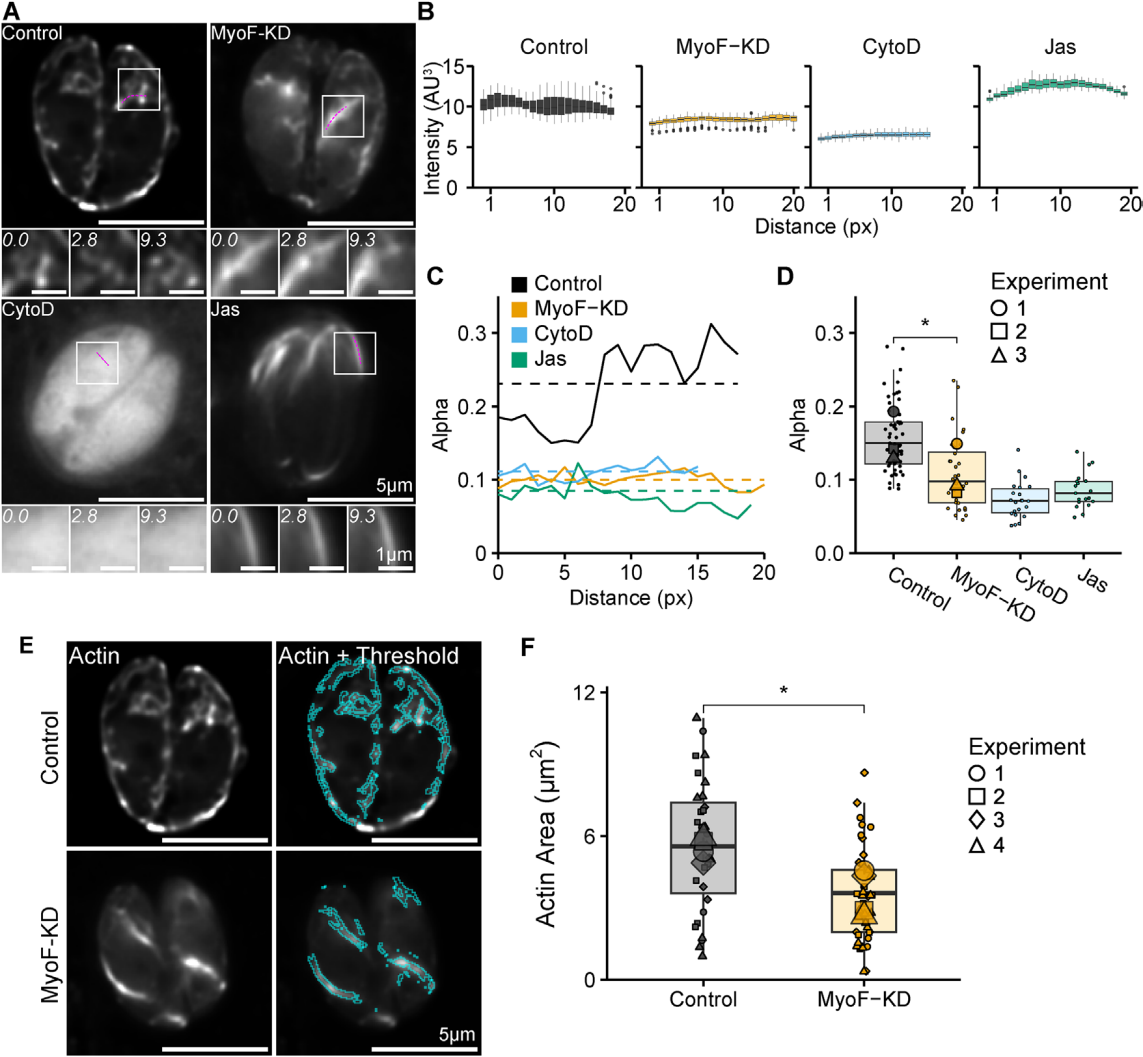
MyoF knockdown alters actin dynamics and organization. Representative images of (A) MyoF-mAID:ActinCB-EmFP parasites treated with either EtOH (control) or IAA (MyoF-KD) for 15 h. After 15 h of treatment, a subset of control parasites were treated with either CytoD or Jas for 60 min before imaging actin organization in live parasites. White box indicates area used to make insets. Time points are indicated in white italics. Dashed magenta lines were used to make fluorescence intensity plots in (B). (B) Boxplots showing pixel-by-pixel changes in fluorescence intensity on magenta lines depicted in (A) in control (grey), MyoF-KD (orange), CytoD (blue), and Jas (green) treated parasites. Boxes represent the interquartile range (IQR) of intensities recorded at each pixel over time, with the bottom and top of the box (hinges) representing the 25^th^ and 75^th^ percentile, respectively. Lines extend to the minimal/maximal value ≤ 1.5 × IQR from their respective hinge. Points indicate outliers beyond 1.5 × IQR. (C) For each pixel on the line an alpha value was calculated (solid lines) as an indicator of variation in fluorescence intensity at each pixel (see *Materials and Methods*). The mean alpha value for each cell is depicted using dashed lines. (D) Box and whisker plots of mean alpha values from cells in three independent experiments in control and MyoF-KD parasites. Large shapes indicate the mean of each experiment. Small dots indicate values from individual cells. Median values for Control and MyoF-KD parasites from three independent experiments were calculated to be 0.153 ± 0.063 and 0.094 ± 0.059, respectively. Asterisk (*) indicates *p* value = 0.019. Significance calculated using a paired *t* test. Median values for CytoD and Jas parasites were calculated to be 0.07 ± 0.028 and 0.08 ± 0.028, respectively. *N* = 53 for Control, 32 for MyoF-KD, 22 for CytoD, and 19 for Jas. (E) *Left.* Actin organization in control and knockdown parasite lines. *Right*. Actin organization overlaid with actin thresholding that was used to calculate actin area (cyan). (F) Box and whisker plots showing actin area from four independent experiments in control (grey) and MyoF-KD (orange) parasites. Large shapes indicate the mean of each experiment. Small shapes indicate values from individual cells. Actin area is significantly reduced upon MyoF-KD. Median values for Control and MyoF-KD parasites from four independent experiments were calculated to be 5.57 ± 0.47 μm^2^ and 3.62± 0.98 μm^2^, respectively. Asterisk (*) indicates *p* value = 0.023. Significance calculated using a paired *t* test. *N* = 31 for Control, 44 for MyoF-KD.

Given that the actin organization in *T. gondii* cannot be described at a single filament level, we sought an alternative approach to quantify the changes in actin dynamics during MyoF depletion. We adapted a method previously developed by (Bronkhorst *et al.*, 2022) that used changes in fluorescence intensity along a line of interest as a readout for actin organization. We reasoned that dynamic actin structures, such as those observed in control parasites, would show a large standard deviation (SD) in fluorescence intensity at any given pixel along the line over time, while more static structures should have a lower deviation in the fluorescence intensity over time. As a proof of concept for quantifying actin dynamics in this way, we treated parasites with the actin-depolymerizing compound Cytochalasin D (CytoD), the actin-stabilizing compound Jasplakinolide (Jas) or ethanol as a control and imaged actin organization in live parasites for 30 s. CytoD-treated parasites exhibited a diffuse signal throughout the cytosol (Figure 4A; CytoD; Supplemental Video S4) while Jas-treated parasites formed large actin masses at both the apical and basal ends of the parasites (Figure 4A; Jas; Supplemental Video S4; Heaslip *et al.*, 2016; Periz *et al.*, 2017). We measured intensity values across the apical cytosol in control and CytoD-treated parasites and the actin bundles of Jas-treated parasites to capture changes in signal intensity over time (Figure 4A, magenta lines; Figure 4B). In the control parasite, the mean intensity was 10.2 × 10^3^ AU ± 1021, while for CytoD and Jas-treated parasites it was 5.3 × 10^3^ AU ± 314 and 12.1 × 10^3^ AU ± 421, respectively. We noted that the mean intensity of the control and Jas-treated parasites were similar, but the fluorescence intensity of actin after Jas stabilization had a lower SD at each pixel. In CytoD-treated parasites, the fluorescence intensity and SD were both lower than controls as expected for a diffuse signal. Next, we used the maximum and mean intensity values for each pixel in the line to calculate an alpha value, a readout for the ratio between maximum and mean intensity values (see *Materials and Methods*; Figure 4C; solid lines). By comparing alpha values of each pixel, we can determine whether the signal across the line was dynamic or static. If the fluorescence intensity does not fluctuate, the maximum and mean values will be equal and result in an alpha value of zero. Dynamic structures, where fluctuations in signal intensity increase the difference between the mean and max intensity values, will have higher alpha values. To obtain a mean alpha value for each cell, we averaged the alpha values at each pixel across the line (Figure 4C; dashed lines). The alpha value for the static actin in CytoD- and Jas-treated parasites were 0.11 and 0.08, respectively, while the alpha values for the control parasites was 0.23, demonstrating the utility of this methodology to quantify fluorescence intensity fluctuation over time as a readout for actin dynamics. To quantify whether there were changes in actin dynamics in MyoF-KD parasites, we measured the fluorescence intensity of actin in the Golgi-adjacent actin masses over time (Figure 4, A–C) and obtained an alpha value of 0.10, similar to that of CytoD- and Jas-treated parasites. Comparing the mean alpha values from three independent experiments, we observed a 30% reduction in the mean alpha value from 0.16 ± 0.03 (mean ± SEM) in control parasites to 0.11 ± 0.04 in MyoF-KD parasites (Figure 4D; *p* value = 0.019). These results indicate that F-actin dynamics in *T. gondii* are dependent on MyoF.

**Figure:** Movie S4 ActinCB‐EmFP expressing parasites in control (top left), MyoF knockdown (top right), CytoD treatment (bottom left), and Jas treatment (bottom right). White box indicates area used to make inset. Imaging speed was 4.9 fps. Playback is 1.5x real time.

In addition to changing actin dynamics, loss of MyoF appeared to change actin organization in the parasite cytosol, with masses of actin adjacent to the Golgi and fewer filaments extending throughout the parasite cytosol. To quantify these changes in actin organization after MyoF depletion, local thresholding was used to segment the actin signal and quantify actin area in the cytosol (Figure 4E). Actin area was measured at each time point of the movies (Supplemental Figure S4; Supplemental Video S5). Because actin area did not show dramatic changes over the course of imaging (Supplemental Figure S4), the measured actin area at each frame were averaged to obtain a mean actin area for each vacuole. By comparing the total actin area between control and MyoF-KD vacuoles across four independent experiments, we observed total actin area decrease by 34% from 5.49 to 3.62 μm^2^ (Figure 4F; *p* value = 0.023). To confirm that the change in actin area was not due to a change in vacuole size upon loss of MyoF, the vacuole area was measured in control and MyoF-KD parasites. No significant change was observed between the two conditions (Supplemental Figure S4C; *p* value = 0.28). Actin area as a ratio of vacuole area decreased by 29% from 0.14 to 0.1 in control and MyoF-KD conditions, respectively (Supplemental Figure S4B; *p* value = 0.037).

**Figure:** Movie S5 Actin area quantification in control (top row) and MyoF-KD (bottom row) parasites. *Left*. ActinCB organization. *Middle*. Fluorescent signal segmented via local thresholding. *Right*. Merge. Imaging speed was 4.9 fps. Playback is 1.5x real time.

**Figure:** Movie S6 MyoF‐KD does not disrupt parasite egress. Control 86 and MyoF‐KD parasites were imaged at five frames per second, with the calcium ionophore A23187 added to induce egress between the first and second frame. Imaging speed was 0.2 fps. Playback is 37.5x real time.

**FIGURE 5:**
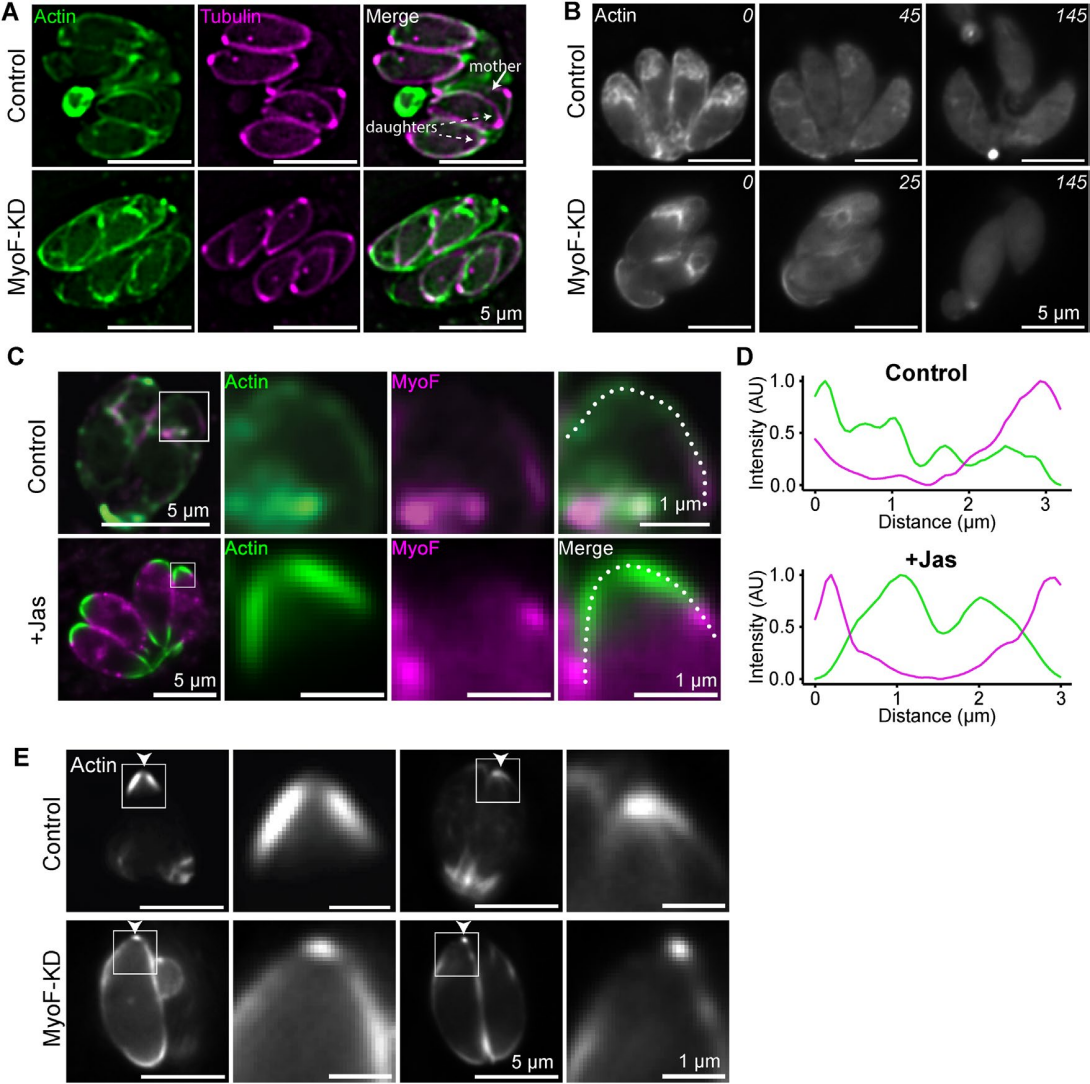
Depletion of MyoF does not affect actin organization during daughter cell construction or parasite egress. (A) Organization of actin (green) and tubulin (magenta) during daughter cell construction. Representative images of MyoF-mAID:ActinCB-EmFP parasites transiently expressing mCherry-TubulinA1 treated with EtOH (control) or IAA (MyoF-KD) and imaged by live cell microscopy during daughter cell construction. Mother and daughter parasites are indicated with arrow and dashed arrow, respectively. In both the presence and absence of MyoF, actin localizes to the daughter cell periphery. (B) Representative images of actin organization during induced parasite egress in control and MyoF-KD parasites. Time in seconds after calcium ionophore treatment is indicated in white italics. In control parasites and upon MyoF depletion, cytosolic actin networks are disassembled and actin accumulation at the parasite’s basal end is observed. (C) Representative images of actin and MyoF organization in control and Jas treated parasites. Parasites expressing ActinCB-EmFP (green) and mCherry-MyoF (magenta) were treated with DMSO or Jas for 60 min before live imaging. White boxes indicate the area used to make insets. Dashed line was used to measure fluorescence intensity of actin and MyoF in panel (D). (D) Normalized fluorescence intensity profile of actin and MyoF in control and Jas treated parasites made using dashed line indicated in (C). Normalization performed via min–max normalization. (E) Two representative images of actin organization in control and MyoF-KD parasites treated with Jas to 60 min prior to imaging. In control parasites, short actin filaments accumulate at the apical and basal ends of the parasite, while in the absence of MyoF actin is found along the length of the parasite periphery. Arrow heads indicate the apical end of the parasite.

During cell division, actin is closely associated with the growing daughter parasites (Periz *et al.*, 2017; Figure 1C). To test whether MyoF knockdown alters actin organization during daughter cell formation, we imaged actin in control and MyoF-KD parasites that were coexpressing mCherry-TubulinA1, which marks the pellicle of both mother and daughter parasites (Figure 5A). In both conditions, actin was strongly enriched at the pellicle of daughter parasites indicating that actin’s association with nascent daughter cells is a MyoF-independent process. Collectively, these data indicate that loss of MyoF results in a change in the dynamics and organization of actin in interphase but not dividing parasites, demonstrating that MyoF is a critical organizer of the actin cytoskeleton in *T. gondii* at this stage of the cell cycle.

### MyoF knockdown does not disrupt parasite egress

*Toxoplasma* actin is essential for a wide range of cellular processes including parasite motility, invasion, and egress. While loss of MyoF caused dramatic changes in actin organization in interphase parasites (Figure 4), MyoF was not required for invasion or egress (Supplemental Video S5; Jacot *et al.*, 2013). Previous studies have shown that actin reorganization coincides with the onset of parasite egress, specifically disassembly of the actin in the RB, followed by a basal accumulation of actin in motile parasites (Periz *et al.*, 2017; Tosetti *et al.*, 2019; Martinez *et al.*, 2023). To determine whether these changes in actin dynamics are affected by loss of MyoF, we imaged actin organization during parasite egress. Control and MyoF-KD parasites were grown overnight and then egress was artificially induced using the calcium ionophore A23187. In control parasites, there was a rapid depolymerization of the actin cytoskeleton, and the ActinCB became diffuse in the cytosol before basal accumulation of actin which coincides closely with the onset of motility (Figure 5B; Supplemental Video S5). Despite the altered actin organization in intracellular interphase parasites in the absence of MyoF, we observed the same sequence of events in MyoF-KD parasites during egress as in control parasites, that is, rapid depolymerization of cytosolic actin, basal accumulation of actin, and successful egress from the host cell. Thus, the altered actin organization and dynamics in interphase parasites in the absence of MyoF did not prevent the rapid reorganization of actin as the parasite switches between nonmotile and motile states, demonstrating that MyoF’s role in actin organization is limited to interphase.

### MyoF enriches to the ends of Jas stabilized F-actin

Given the role of MyoF in regulating actin organization, we investigated whether the localization of MyoF was affected by Jas treatment. Jas results in the formation of static bundle-like actin structures at the apical and basal ends of the parasite (Figure 5C). To observe the localization of MyoF during Jas stabilization, a second copy of MyoF (pMyoF-mCherry-MyoF) was expressed from the dispensable UPRT locus of ActinCB expressing parasites. In Jas-treated parasites, there was a reduction in cytosolic MyoF compared with controls while the peripheral signal of MyoF was still present (Figure 5C). A punctum of MyoF was localized at the basal end of these actin structures. (Figure 5, C and D).

### MyoF knockdown alters Jas-induced actin stabilization

It is unclear why actin localizes to the apical and basal ends of the parasites upon Jas treatment but is potentially due to the action of one or more actin-binding proteins. We were intrigued by the observation that MyoF localized adjacent to the bundle-like actin structures after Jas treatment, so we investigated whether MyoF influenced actin organization in Jas-treated parasites. MyoF-mAID:ActinCB-EmFP parasites were treated with either EtOH or IAA overnight then Jas treated for 60 min before imaging. In control parasites, F-actin was restricted to the apical and basal ends of the parasite, as expected. However, in the absence of MyoF, F-actin extended along the parasite periphery, indicating that MyoF also influences actin organization in Jas-treated parasites. (Figure 5E).

## DISCUSSION

Intracellular trafficking is a vital cellular process that is necessary for *T. gondii* survival and pathogenicity. Actin and MyoF are necessary for vesicle transport and the organization of compartments in the endomembrane pathway. The central goal of this study was to further understand how MyoF and F-actin facilitate these essential cellular processes. We began by comparing the dynamics of actin and MyoF in live parasites. Expression of the ActinCB was used for imaging actin organization in *T. gondii* as it is the only methodology that is tolerated by live parasites. (Periz *et al.*, 2017). Overexpression of the chromobody at high levels can alter actin dynamics, however we demonstrate that stable expression of the chromobody at low levels does not disrupt parasite viability, consistent with previous studies (Periz *et al.*, 2017). MyoF and F-actin have a highly dynamic, filamentous-like organization in the cytosol. Using live cell imaging of F-actin, MyoF and cargo, we find that MyoF is essential for the organization of the compartments but does not stably associate with them. Instead, both MyoF and actin dynamically surround the apicoplast during the elongation phase of apicoplast division. In addition, motile dense granules did not move linearly along actin filaments, instead vesicles appeared to move in concert with dynamic actin masses, indicating that MyoF likely transports cargo using a noncanonical transport mechanism. Consistent with this, we find that MyoF is needed to power a highly dynamic F-actin cytoskeleton undergoing constant rearrangement. Collectively, the data indicates that an alternative transport mechanism exists in *T. gondii* which is actin-focused and relies on MyoF for its movement.

MyoF is part of a class of myosin motors found exclusively in Apicomplexan parasites and is the only myosin class to contain a WD40 domain at its C-terminus. A number of actin binding proteins such as coronin bind actin via their WD40 domains (Salamun *et al.*, 2014; Olshina *et al.*, 2015). This led us to speculate that MyoF functions in actin organization by dynamically crosslinking filaments through its motor and tail domains. Thus, to determine whether MyoF functions as an actin organizer, we imaged the organization of actin in *T. gondii* depleted for MyoF. We find that loss of MyoF results in dramatic changes in both the dynamics and organization of actin within the cytosol of interphase parasites. In control parasites, actin filaments emanate throughout the apical end of the cytosol and undergo continual rearrangement. Upon depletion of MyoF, large bundle-like masses of actin accumulate adjacent to the Golgi. Although some filaments can be seen escaping these masses (Supplemental Video S4), the organization of actin in MyoF depleted cells is static compared with actin filaments in control parasites. This data led us to propose the following model for how MyoF facilitates cargo transport: Rather than binding and carrying cargo directly, MyoF controls the organization, and dynamics of actin by dynamically crosslinking and dispersing actin filaments nucleated by Golgi-associated formin-2. Endomembrane cargos are then transported by associating with the dynamic actin network through vesicular cargo linkers that are yet to be identified. The mechanism by which MyoF mediates this filament dispersion requires investigation in a future study.

During canonical cargo transport, molecular motors serve as a linker between cargo and the filamentous cytoskeleton, but the mechanism for linking membranous cargo to the MyoF-actin network in *T. gondii* is currently unclear. While we do not observe MyoF stably associating with dense granules or the apicoplast, we cannot rule out that interactions between MyoF and cargo occur transiently. A recent study by (Bibeau *et al.*, 2020) in *Physcomitrella patens* demonstrated that myosin XI associates with filaments and contributes to vesicle transport through transient (0.5–2 s) vesicle interactions.

In addition to the dynamic actin filaments observed in interphase parasites, *Toxoplasma* actin forms additional structures during the parasite’s lytic cycle. During daughter cell development, actin surrounds the growing daughter parasite (Figure 1B; Periz *et al.*, 2017). Actin in the RB connects intracellular parasites during replication (Periz *et al.*, 2017; Hunt *et al.*, 2019), while peripheral actin drives parasite motility, invasion, and egress. MyoF was not required for the organization of actin in these structures, which is consistent with these cellular processes being unaffected by the loss of MyoF. Higher resolution characterization of actin organization was not undertaken due to the changes in actin structure that occur upon cell fixation, a prerequisite for most superresolution microscopy techniques. We speculate that changes are due to partial depolymerization of actin filaments before fixation goes to completion, a phenomenon that has previously been observed in other cell types (Geron *et al.*, 2013).

Mechanisms of actin organization in Apicomplexa are poorly understood. A number of actin binding proteins have been characterized in *T. gondii* including formin, profilin, CAP, and ADF (Schüler and Matuschewski, 2006; Mehta and Sibley, 2011; Skillman *et al.*, 2012; Hunt *et al.*, 2019; Das *et al.*, 2021), all of which regulate actin filament nucleation and turnover. In addition, *T. gondii* contains a single coronin gene which may crosslink actin filaments but loss of this protein did not affect actin-based cellular processes or parasite viability (Salamun *et al.*, 2014). Thus, MyoF is the first protein identified in *T. gondii* which regulates actin filament organization. Future studies will focus on elucidating the mechanism utilized by MyoF to regulate actin organization.

## MATERIALS AND METHODS

Request a protocol through *Bio-protocol*.

### Cell culture and parasite transfection

#### HFF and *T. gondii* culture.

All *T. gondii* lines used in this study were derived from the RH strain. Parasites were cultured via continuous passage in confluent human foreskin fibroblasts (HFFs) grown in DMEM containing 1X antibiotic/antimycotic and 1% (vol/vol) heat-inactivated fetal bovine serum (FBS) at 37°C with 5% CO_2_. HFFs were grown in DMEM containing 1X antibiotic/antimycotic and 10% (vol/vol) heat-inactivated FBS at 37°C with 5% CO_2_.

#### Parasite transfection.

All parasite transfections were carried out as previously described in (Roos *et al.*, 1995) using a BTX electroporator with set to 1500 V, 25 Ω, and 25 μF. For transient transfections, 25 μg of plasmid was electroporated into 1 × 10^7^ parasites, after which parasites were placed into MatTek dishes (MatTek Corporation, Ashland, MA) containing confluent HFF monolayers and allowed to grow overnight before imaging. For CRISPR/Cas9 genome editing, 5 μg of plasmid containing a Cas9 expression cassette and guide RNA were cotransfected with 50 μl of homologous recombination oligomer amplified by PCR (Sidik *et al.*, 2014; O’Shaughnessy *et al.*, 2020).

#### Drug treatment.

To knock down the expression of MyoF; MyoF-mAID:ActinCB-EmFP parasites were allowed to invade confluent HFF monolayers for 1 h. Parasites were then grown in IAA at a final concentration of 500 μM or an equivalent volume of 100% ethanol (vehicle) for 15 to 18 h. To perturb actin dynamics, parasites were treated with either 5 μM CytoD or 1 μM Jas for 60 min before imaging.

#### Fluorescence-activated cell sorting (FACS).

For isolating clonal lines based on expression of fluorescent proteins, transfected parasites were harvested after lysis of the HFF monolayer (∼48 h posttransfection) and passed through a 16-gauge needle to disrupt parasite aggregates. Syringe-released parasites were then filtered through a 35-μm cell strainer cap attached to a 5 ml tube. FACS was performed using a BD FACS Aria II Cell Sorter to sort parasites into a 96-well plate containing confluent HFF monolayers to establish clonal lines.

#### Plaque assay.

To determine whether integration of the ActinCB effected parasite viability, 200 MyoF-mAID parasites and MyoF-mAID:ActinCB-EmFP parasites were plated into a six-well plate containing confluent HFF monolayers and grown in the presence of 500 μM IAA (Sigma-Aldrich; I2886) or an equivalent volume of 100% EtOH. After plating, parasites were allowed to grow undisturbed for 11 d. Cells were fixed with –20°C methanol for 5 mins followed by staining with Coomassie (40% methanol, 7.5% acetic acid, 0.025% Coomassie Brilliant blue R 250, and 52.5% H_2_O) at room temperature for 2 h. Cells were then washed multiple times with water to remove excess Coomassie stain.

#### Fixed cell imaging of ActinCB expressing parasites.

To determine whether fixation disrupted the actin cytoskeleton, ActinCB expressing parasites were fixed with multiple methodologies and compared with live parasites. The fixation procedures tested were as follows: (A) parasites treated with 0.3% glutaraldehyde (Electron microscopy sciences, Catalogue #16000) for 1 min, then 0.25% TX-100 for 1 min, then 0.1% NaBH_4_ for 7 min. (B) parasites treated with 0.3% glutaraldehyde for 1 min, then 0.25% TX-100 for 1 min, then 2% glutaraldehyde for 15 min, then quenched with 0.1% NaBH_4_ for 7 min. (C) parasites treated with freshly made 4% paraformaldehyde (PFA) (Electron microscopy sciences, Catalogue #15714) for 40 min, then 0.25% TX-100 for 1 min. (D) parasites were treated simultaneously with 0.3% glutaraldehyde and 0.25% TX-100 for 1 min, then quenched with 0.1% NaBH_4_ for 7 min. (E) parasites treated simultaneously with 0.3% glutaraldehyde and 0.25% TX-100 for 1 min, then 2% glutaraldehyde for 15 min, then quenched with 0.1% NaBH_4_ for 7 min. (F) parasites treated with 4% PFA for 30 min. All fixatives were made with 1xPBS. After fixation, all parasites were washed with 1xPBS and imaged (Thermo Fisher Scientific; 18912–014; Supplemental Figure S3).

### Construction of expression plasmids and parasite lines

A list of plasmids, primers, and gene accession numbers used in this study can be found in Supplemental Tables S1, S2, and S3, respectively.

#### Creation of pMIN-ActinCB-EmFP.

pMIN-ActinCB-tdTomatoFP-CAP plasmid (kind gift from Dr. Michael Reese, UT Southwestern Medical Center) was digested with NheI/XbaI to remove ActinCB-tdTomatoFP. The ActinCB-EmFP coding sequence was amplified via PCR using primers (F1 and R1) to add 40 bp of homology complementary to the digested vector. The digested vector and PCR amplicon were combined through Gibson assembly (Gibson *et al.*, 2009) using a custom Gibson assembly master mix (McAllaster *et al.*, 2016) to create pMIN-ActinCB-EmFP. The plasmid was transformed into NEB5α bacteria and positive clones screened by colony PCR and verified via Sanger sequencing.

#### Creation of MyoF-mAID:ActinCB-EmFP parasite line.

To create a parasite line stably expressing ActinCB-EMFP from the Ku80 locus under the control of the minimal DHFR promoter, a homologous recombination oligo (HR-oligo) was created by PCR by amplifying the pMIN-ActinCB-EmFP plasmid with primers F1 and R1. In addition to the HR oligo, a plasmid containing a Cas9-expression cassette and guide-RNA targeting the 5′UTR of the Ku80 locus (kind gift from Dr. Michael Reese; O’Shaughnessy *et al.*, 2020) was cotransfected via electroporation into 1 × 10^7^ *ΔΚu80:ΔHXGPRT:FLAG-TIR1:MyoF-mAID-HA* parasites (Carmeille *et al.*, 2021) and plated into a T-25 containing a confluent host monolayer. After lysis of the monolayer, parasites were sorted using FACS into a 96-well plate as described above to isolate clonal lines. Genomic DNA was isolated from clonal lines and amplified by PCR using primers F1 and R1 to verify the integration of the ActinCB-EmFP expression cassette into the Ku80 genomic locus (Supplemental Figure S2). A plaque assay was used to confirm no deleterious effects on parasite viability due to integration of the chromobody (Supplemental Figure S1B).

#### Creation of mCh-MyoF:ActinCB-EmFP parasite line.

To visualize both MyoF and actin in live parasites simultaneously, pMyoF-mCherry-MyoF (Devarakonda *et al.*, 2023), which contains the MyoF coding sequence from TgME49_278870 (Amos *et al.*, 2022), was amplified with homologous overhangs to the *UPRT* locus with primers F3 and R3 and cotransfected with the pSAG1::CAS9-U6::sgUPRT (Shen *et al.*, 2014; Addgene #54467) into RHΔKu80ΔHXGPRT parasites. After lysis of the monolayer, parasites were sorted using FACS as described above to isolate clonal lines. Genomic DNA was isolated from clonal lines and amplified by PCR using primers F2 and R2 to verify the integration of pMyoF-mCherry-MyoF into the *UPRT* locus (Supplemental Figure S1). Once the mCherry-MyoF parasite line was established, ActinCB-EmFP was integrated into the Ku80 locus of mCherry-MyoF parasites as described above to create the mCherry-MyoF:ActinCB-EmFP parasite line.

### Microscopy

Imaging was performed using a DeltaVision Elite microscope system built on an Olympus base with a 100 × 1.39 NA objective in an environment chamber heated to 37°C. The system utilizes a scientific CMOS camera and DV Insight solid state illumination module with the following excitation wavelengths: DAPI = 390/18 nm, FITC = 475/28 nm, TRITC = 542/27 nm, and Alexa 647 = 632/22 nm. Single band pass emission filters had the following wavelengths: DAPI 435/48 nm, FITC = 525/48 nm, TRITC = 597/45 nm, and Alexa 647 = 679/34 nm. Image acquisition speeds were determined on a case-by-case basis as noted in the video legends. For fixed-sample imaging, Z-stacks were acquired with a z-step of 0.2 µm. Live cell imaging was performed on a single focal plane.

#### Preparing live cell imaging samples.

For live cell imaging of parasites, 5 × 10^4^ parasites were plated into each MatTek dish containing a confluent HFF monolayer and allowed to grow for 15–18 h (overnight). The next day, media from the MatTek dishes was aspirated, and the dishes were washed three times using Fluorobrite DMEM warmed to 37°C (Thermo Fisher Scientific, A1896701) supplemented with 1% FBS, 1X antibiotic/antimycotic, and any applicable drug, with a 5-min incubation at 37°C before the final wash and imaging.

#### Two color imaging of MyoF and Actin with microtubule, Golgi, apicoplast, and dense granule markers.

For visualizing MyoF and actin with markers for microtubules, the Golgi, apicoplast, or dense granules, MyoF-EmFP parasites and MyoF-mAID:ActinCB-EmFP parasites were transiently transfected with 25 μg of plasmids containing the following expression cassettes: pTub-GRASP-mCherry to mark the Golgi (Pelletier *et al.*, 2002), pTub-FNR-RFP to mark the apicoplast, (Vollmer *et al.*, 2001), pTub-SAG1ΔGPI-mCherry to mark the dense granules (Heaslip *et al.*, 2016) or pTub-mCherry-TubulinA1 (Hu *et al.*, 2002) to mark the microtubule cytoskeleton. The transfected parasites were then cultured overnight on confluent HFF monolayers in MatTek dishes before performing live-cell imaging in Fluorobrite DMEM as described above. Imaging frame rates were determined on a frame-by-frame basis and are noted in the figure legends. Representative images shown in figure 2 were chosen from 75 vacuoles (Golgi; four independent experiments), 114 vacuoles (apicoplast; six independent experiments) and 46 vacuoles (dense granules; three independent experiments).

#### Imaging MyoF-mAID:ActinCB-EmFP parasites during Jas and CytoD treatment.

To visualize chemical perturbation of the actin cytoskeleton, MyoF-mAID:ActinCB-EmFP parasites were treated with either 500 μM IAA or an equivalent volume of ethanol and grown for 15–18 h in confluent HFF monolayers in MatTek dishes. Before imaging, media from the MatTek dishes was aspirated, and the dishes were washed three times using 37°C Fluorobrite DMEM (Thermo Fisher Scientific, A1896701) supplemented with 1% FBS, 1X antibiotic/antimycotic, and either 500 μM IAA or an equivalent volume of ethanol, with a 5-min incubation at 37°C before the final wash. After washing, CytoD or Jas was added to a final concentration of 5 μM and 1 μM, respectively, or an equivalent volume of DMSO. The parasites were incubated in drug for 60 min before imaging.

#### Imaging MyoF-mAID:ActinCB-EmFP parasites ±/- IAA during egress.

To visualize the organization of actin during egress in control and MyoF knockdown parasite lines, MyoF-mAID:ActinCB-EmFP parasites were grown in the presence of ethanol or IAA as described above. To image parasites during egress, the calcium ionophore A23187 (Sigma-Aldrich; C7522) was added to a final concentration of 5 μM between the first and second frame of the time-lapse images, and imaging was allowed to proceed until parasites egressed.

### Image processing and analysis

#### Deconvolution.

Deconvolution was performed using SoftWorx v7.2.2(Conservative ratio, four iterations). All images used for figures are deconvolved unless stated otherwise. For intensity quantification, nondeconvolved images were used for measurements.

#### Bleach correction.

All time-lapse images were bleach corrected using the histogram matching method in FIJI (Schindelin *et al.*, 2012; Miura, 2020)

#### Kymographs.

To create kymographs of MyoF, actin, and dense granules, line segments were drawn over dense granule movement events and used to create kymographs using the Reslice function in FIJI.

#### Quantification of MyoF and Actin localizations.

To quantify the percentage of parasites exhibiting cytosolic, peripheral, and RB MyoF or actin signal, the following criteria for each region were used: cytosolic contained signal primarily originating apical of the Golgi, peripheral contained signal appearing distinctly along the periphery of the parasite, RB contained signal distinct from either parasite’s cytosol or periphery and localized to the basal end of the vacuole.

#### Quantification of actin dynamics.

To quantify the changes in the dynamics of actin during CytoD treatment, Jas treatment, and MyoF knockdown, time-lapse images of MyoF-mAID:ActinCB-EmFP parasites treated with ethanol or IAA were acquired as described above across three independent experiments (*n* = 53 ethanol, 32 IAA, 22 CytoD, 19 Jas). Line segments were manually drawn over the center of apical actin in –IAA images, over the largest cytosolic mass of actin in +IAA parasites, over the apical cytosol in CytoD-treated parasites, and over the apical periphery actin in Jas-treated parasites using the line segment tool in FIJI. The fluorescence intensity along the line for each frame of the movie was measured using the Plot Profile tool in FIJI to collect the intensity values at each pixel. For each pixel along the line, we calculated alpha (α), a measure of the variability in the fluorescence intensity, by dividing the highest intensity value (I_max_) by the mean intensity value (I_mean_). 1 was subtracted from the resulting value.




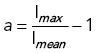




In instances of little change in fluorescence intensity over time, the maximum and mean intensities will be similar, meaning the ratio (alpha) will be close to zero. In instances of large fluctuations in fluorescence intensity over time, the difference in maximum and mean intensity values will increase, resulting in a higher alpha value. For statistical comparison between treatments, the mean alpha value of all parasites within each treatment-experiment pair was used to perform a student’s *t* test.

#### Quantification of actin area.

To quantify the changes in actin area during MyoF knockdown, time-lapse images of MyoF-mAID:ActinCB-EmFP treated with ethanol or IAA were analyzed. Vacuoles that met the following criteria were analyzed: contained two interphase parasites, both parasites appeared to be at the same Z-depth, and there was no PV actin signal above or below the parasite cytosol (*n* = 31 ethanol, 44 IAA, four independent experiments). Analysis was performed on deconvolved and bleach corrected images in FIJI. To determine the ratio of actin area to vacuole area, the entire vacuole was first outlined using thresholding and manual adjustment to create an inverted mask. This mask was saved for use after thresholding the actin signal to remove segmentation artifacts outside of the vacuole. The original image was then processed in FIJI using standard thresholding methods. The images were converted from 16-bit to 8-bit, a Gaussian blur (σ = 2) was applied, and then local thresholding (Bernsen algorithm, radius = 1) was performed on each frame to create a binary image stack of actin area over time. The inverted vacuole mask was then used to mask any segmentation artifacts outside of the parasites and measure the total vacuole area (Supplemental Figure S4). If any noncytosolic actin signal was still present, the remaining artifacts were manually removed. After removing artifacts, the cytosolic actin area was measured at each frame of the movie and used to calculate the mean actin area of each vacuole (Supplemental Figure S4). To control for differences in parasite size, the mean actin area of each image stack was divided by the vacuole area to determine the proportion of the vacuole containing actin filaments. For statistical comparison, a paired student’s *t* test was performed on the mean values from each treatment-experiment pair.

### Analysis software

Image measurements, segmentation, and processing was done using FIJI (v1.54f). Data analysis was done using R Statistical Software (v4.3.1). Data was handled and visualized using the tidyverse package (v2.0.0). Statistics were calculated and visualized using the ggpubr package (v0.6.0).

## Supplementary Material



## Abbreviations used:

ActinCB: Actin chromobody
PV: parasitophorous vacuole
RB: Residual body.
