# Supplementary material for: Myosin F controls actin organization and dynamics in Toxoplasma gondii
Source: Mol Biol Cell. 2024 Mar 12;35(4):ar57. doi: 10.1091/mbc.E23-12-0510 (PMC11064658; doi:10.1091/mbc.E23-12-0510)
Supplement: Supplementary file 7 [file mbc-35-ar57-s001.pdf]

Supplementary Materials

Molecular Biology of the Cell

Kellermeier and Heaslip

Myosin F controls actin organization and dynamics in *Toxoplasma gondii*

Jacob A. Kellermeier and Aoife T. Heaslip*

Supplemental Materials

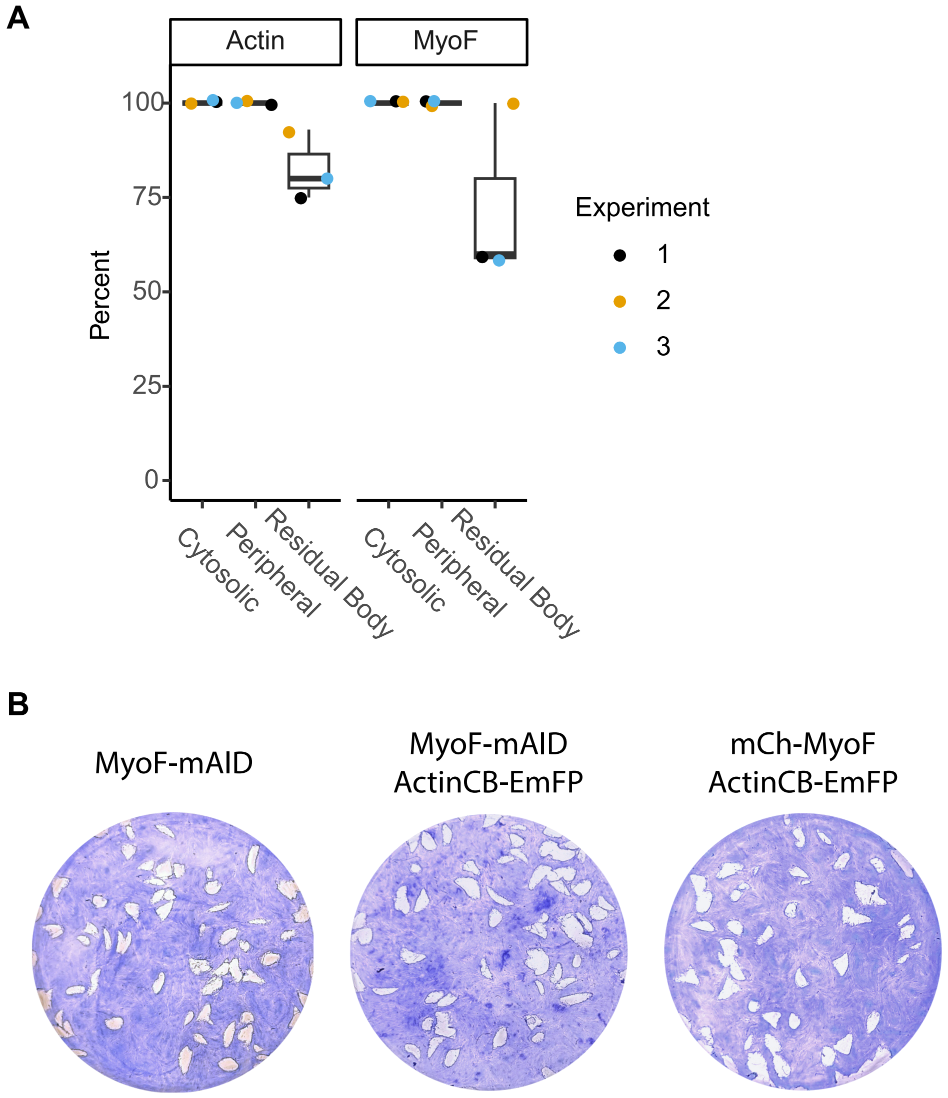
A
Actin
MyoF
100
75
50
25
0
Percent
Cytosolic
Peripheral
Residual Body
Cytosolic
Peripheral
Residual Body
Experiment
1
2
3
B
MyoF-mAID
MyoF-mAID
ActinCB-EmFP
mCh-MyoF
ActinCB-EmFP

Figure S1.

Figure S1. (A) Percentage of vacuoles with ActinCB-EmFP and MyoF-EmFP in the cytosol, periphery, and residual body. The criteria for each region were as follows: cytosolic contained signal primarily originating apical of the Golgi, peripheral contained signal appearing distinctly along the periphery of the parasite, residual body contained signal distinct from either parasite's cytosol or periphery, and outside of the parasite cytosol. Mean values from three independent experiments were calculated. Residual body localization of ActinCB-EmFP and MyoF-EmFP was found in 83 ± 9.3% and 73 ± 24% of parasites, respectively. N = 46 ActinCB-EmFP, 84 MyoF-EmFP parasites. (B) Plaque assays of MyoF-mAID, MyoF-mAID:ActinCB-EmFP, and mCh-MyoF:ActinCB-EmFP parasites. Integration of the chromobody into the MyoF-mAID parental line does not prevent plaque formation in control lines or MyoF knockdown-induced lethality. Expression of a second copy of MyoF from the dispensible *UPRT* locus and integration of the chromobody does not prevent plaque formation.

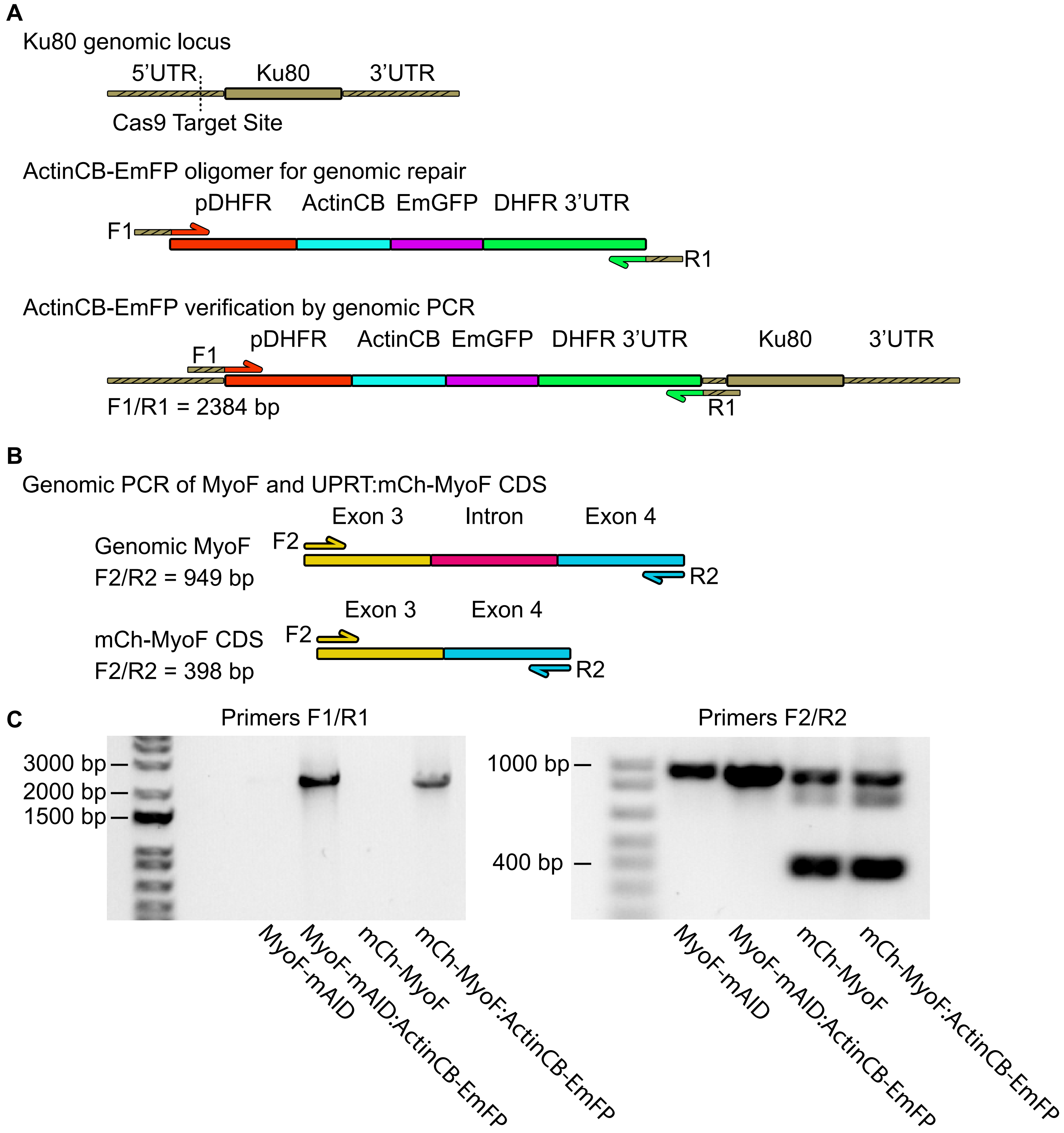
A
Ku80 genomic locus
5'UTR
Ku80
3'UTR
Cas9 Target Site
ActinCB-EmFP oligomer for genomic repair
pDHFR
ActinCB
EmGFP
DHFR 3'UTR
F1
R1
ActinCB-EmFP verification by genomic PCR
F1
pDHFR
ActinCB
EmGFP
DHFR 3'UTR
Ku80
3'UTR
F1/R1 = 2384 bp
R1
B
Genomic PCR of MyoF and UPRT:mCh-MyoF CDS
Exon 3
Intron
Exon 4
Genomic MyoF
F2/R2 = 949 bp
F2
R2
Exon 3
Exon 4
mCh-MyoF CDS
F2/R2 = 398 bp
F2
R2
C
Primers F1/R1
3000 bp
2000 bp
1500 bp
Primers F2/R2
1000 bp
400 bp
MyoF-mAID
MyoF-mAID:ActinCB-EmFP
mCh-MyoF
mCh-MyoF:ActinCB-EmFP
MyoF-mAID
MyoF-mAID:ActinCB-EmFP
mCh-MyoF
mCh-MyoF:ActinCB-EmFP

Figure S2.

Figure S2. (A) Diagrams of Ku80 locus in parental parasites, and after integration of ActinCB-EmFP expression cassette into the Ku80 locus via CRISPR/Cas9. Binding sites of primers used to integrate the ActinCB cassette and verify its integration are indicated. (B) Diagrams of a portion of the MyoF genomic locus and after integration of pMyoF-mCherry-MyoF coding sequence (CDS) into the *UPRT* locus. Binding sites of primers used to differentiate genomic MyoF and MyoF CDS are indicated. (C) *Left*. Genomic PCR verification of ActinCB-EmFP integration into the Ku80 locus of MyoF-mAID parasites and pMyoF-mCherry-MyoF parasites. *Right.* PCR showing integration of pMyoF-mCherry-MyoFCDS into the UPRT locus. Primers binding sites are indicated in A and B respectively. All parasite lines amplify the genomic *MyoF* locus, while only parasites containing the MyoF CDS in the *UPRT* locus amplify a second smaller product.

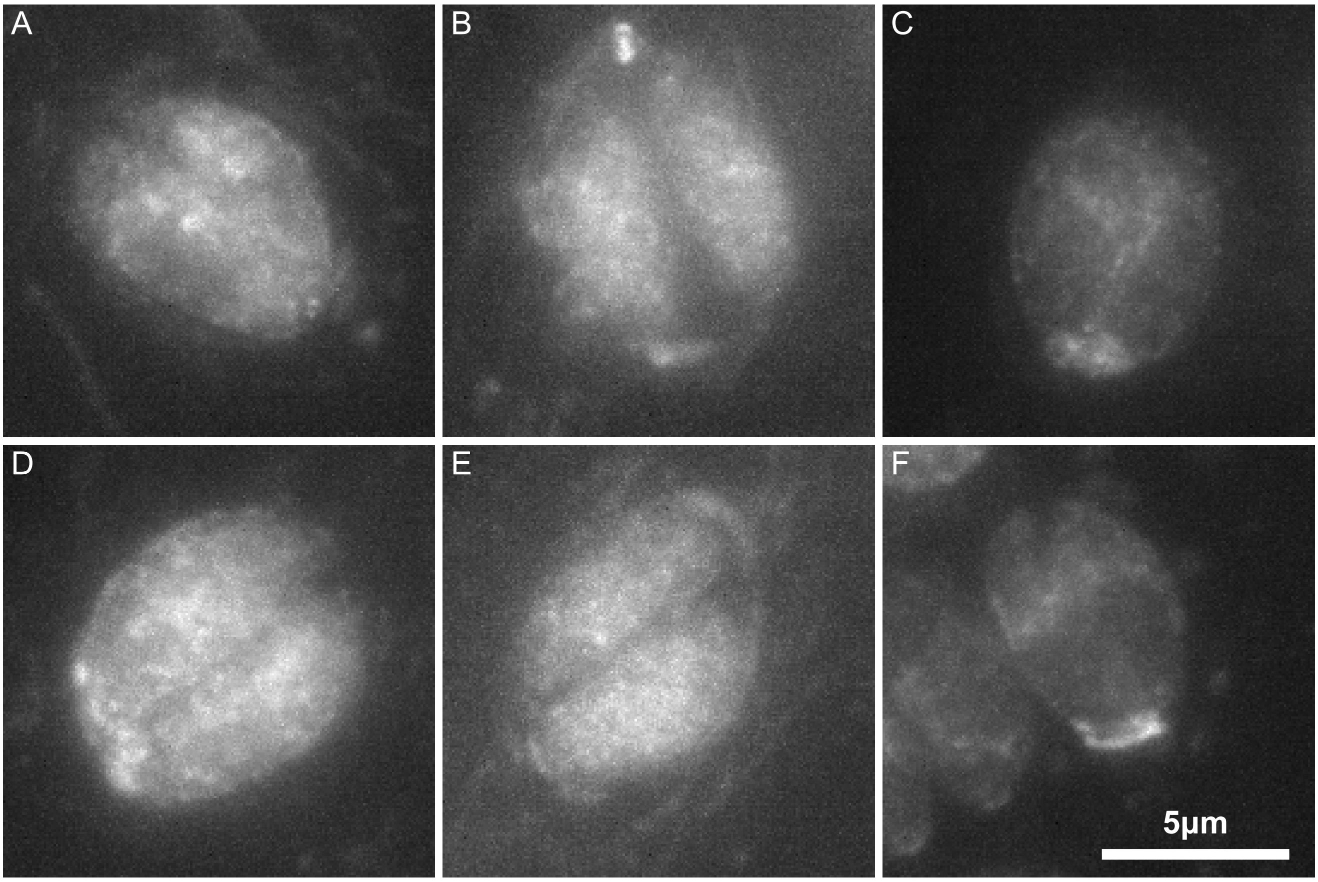

Figure S3.

Figure S3. Effect of fixation on actin organization. MyoF-mAID:ActinCB-EmFP parasites were fixed using multiple conditions to determine fixation preserve the cytosolic actin organization. After fixation, all parasites were washed with 1xPBS and imaged. (A) Parasites treated with 0.3% glutaraldehyde for 1 minute, then 0.25% TX-100 for 1 minute, then 0.1% $NaBH_4$ for 7 minutes. (B) Parasites treated with 0.3% glutaraldehyde for 1 minute, then 0.25% TX-100 for 1 minute, then 2% glutaraldehyde for 15 minutes, then quenched with 0.1% $NaBH_4$ for 7 minutes. (C) Parasites treated with 4% PFA for 40 minutes, then 0.25% TX-100 for 1 minute. (D) Parasites were treated simultaneously with 0.3% glutaraldehyde and 0.25% TX-100 for 1 minute, then quenched with 0.1% $NaBH_4$ for 7 minutes. (E) Parasites treated simultaneously with 0.3% glutaraldehyde and 0.25% TX-100 for 1 minute, then 2% glutaraldehyde for 15 minutes, then quenched with 0.1% $NaBH_4$ for 7 minutes. (F) Parasites treated with 4% PFA for 30 minutes. In all conditions, ActinCB signal was diffuse with no discernable actin filaments.

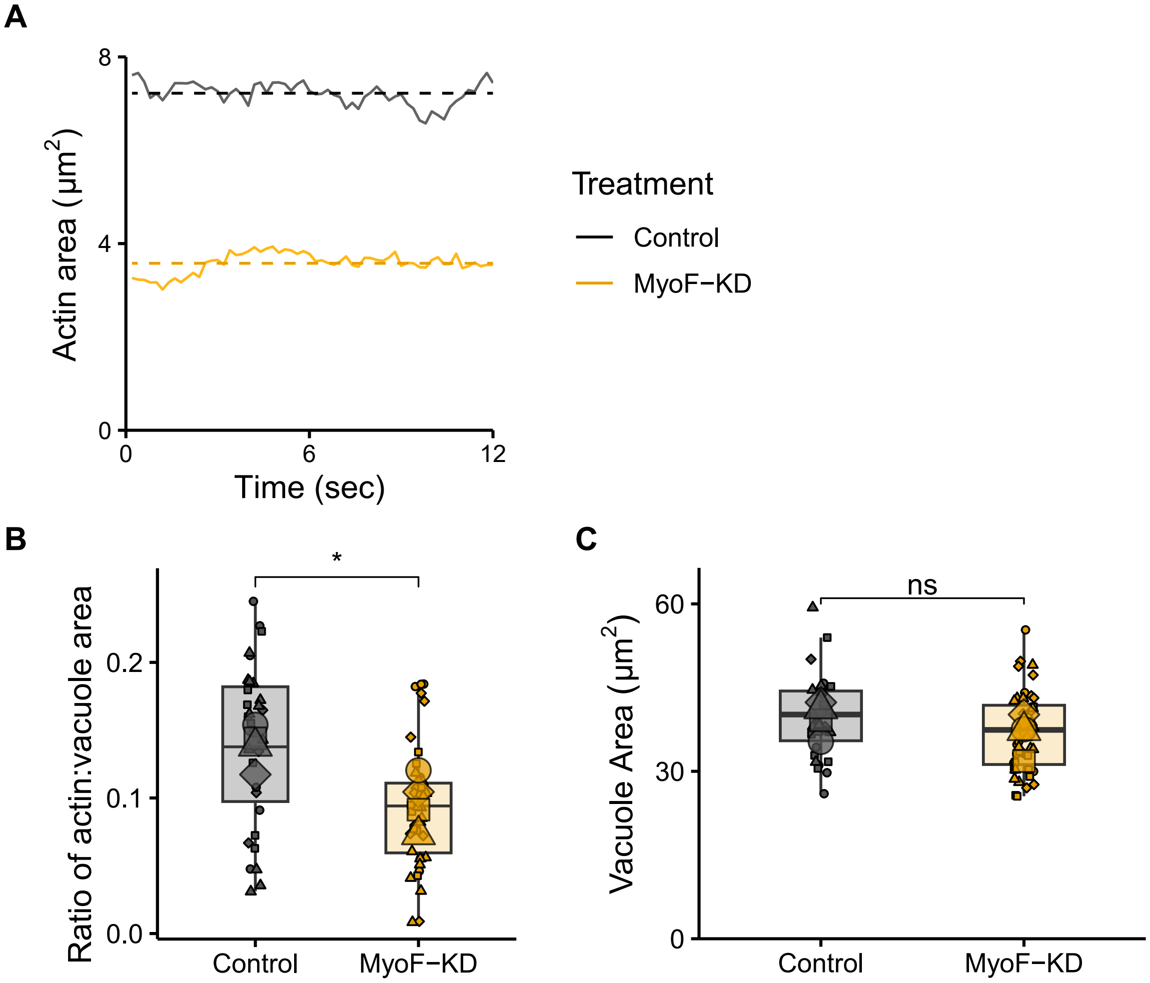
A
8
4
0
Actin area (μm2)
0
6
12
Time (sec)
Treatment
Control
MyoF−KD
B
*
Ratio of actin:vacuole area
0.2
0.1
0.0
Control
MyoF−KD
C
ns
60
30
0
Vacuole Area (μm2)
Control
MyoF−KD

Figure S4.

Figure S4. (A) In control (black) and MyoF-KD (orange) parasites the calculated actin area did not change significantly over the course of imaging. Solid lines represent the measured actin area of parasites in Fig. 4E at each frame of the videos. Imaging speed was 5 frames/second. Dashed lines represent the mean actin area of each video. (B) Box and whisker plots showing ratio of actin area to vacuole area from four independent experiments in control (grey) and MyoF-KD (orange) parasites. Large shapes indicate the mean from each experiment. Small shapes indicate values from individual cells. Median values for Control and MyoF-KD parasites from 4 independent experiments were calculated to be 0.141 ± 0.015 and 0.098 ± 0.02, respectively. Asterisk (*) indicates p-value = 0.037. Significance calculated using a paired t-test. N = 31 Control, 44 MyoF-KD. (C) Vacuole area did not change between Control and MyoF-KD parasites. Median values for Control and MyoF-KD parasites from 4 independent experiments were calculated to be 40.17 ± 3.11 μm^2 and 37.42 ± 3.50 μm^2, respectively. Non-significance (ns) indicates p-value = 0.28. Significance calculated using a paired t-test. N = 31 Control, 44 MyoF-KD.

Supplemental Video Legends.

Video S1: *Top row*. MyoF-EmFP (*left*) and ActinCB-EmFP (*right*) organization during interphase Insets indicate cytosolic, peripheral, and residual body signal. Imaging speeds were 2.5 and 4.9 fps, respectively. Playback is 3x and 6x real time. *Bottom row*. Bottom panels show MyoF-EmFP (yellow; left) and MyoF-mAID:ActinCB-EmFP (Green; right) parasites transiently expressing pTub-mCherry-TubulinA1 (magenta) to mark the tubulin cytoskeleton of daughter parasites. Insets show MyoF and actin localized to the growing daughter cells. Imaging speeds were 1 and 1.5 fps, respectively. Playback is 7.5x and 4.85x real time.

Video S2: MyoF-EmFP (yellow) and MyoF-mAID:ActinCB-EmFP (green) parasites transiently expressing pTub-GRASP-mCherry (top row; magenta), pTub-FNR-RFP (middle row; magenta), or pTub-SAG1ΔGPI-mCherrry (bottom row; magenta). Top show shows MyoF and actin closely associated with the apical face of the Golgi. Imaging speed was 1 fps. Playback is 15.7x real time. Middle panels show MyoF and actin dynamically localized along the apicoplast during elongation. Imaging speed was 1.7 and 1.5 fps, respectively. Playback is 9x and 9.9x real time. Bottom panels show dynamics of MyoF and actin during directed dense granule motion events. Imaging speed was 1.3 and 1.8 fps, respectively. Playback is 5.65 x and 4.15x real time.

Video S3: MyoF-mAID:ActinCB-EmFP parasites transiently expressing GRASP-mCherry in control and MyoF-KD parasites. Top panels show actin dynamically associated with the apical face of the Golgi. Bottom panels show large masses of actin tightly associated with the Golgi. White box indicates area used to make inset panels. Imaging speed was 1 fps. Playback is 7.85x real time.

Video S4: ActinCB-EmFP expressing parasites in control (top left), MyoF knockdown (top right), CytoD treatment (bottom left), and Jas treatment (bottom right). White box indicates area used to make inset. Imaging speed was 4.9 fps. Playback is 1.5x real time.

Video S5: Actin area quantification in control (top row) and MyoF-KD (bottom row) parasites. *Left*. ActinCB organization. *Middle*. Fluorescent signal segmented via local thresholding. *Right*. Merge. Imaging speed was 4.9 fps. Playback is 1.5x real time.

Video S6: MyoF-KD does not disrupt parasite egress. Control and MyoF-KD parasites were imaged at five frames per second, with the calcium ionophore A23187 added to induce egress between the first and second frame. Imaging speed was 0.2 fps. Playback is 37.5x real time.

Supplemental Tables

Table S1: List of plasmids used in this study.

Plasmid Name	Purpose	Reference
pTub-mCherry-TubulinA1	Fluorescent labeling of parasite tubulin	Hu et al., 2002; PMID: 11901169
pUPRT-pMyoF-mCherry-MyoFCDS-3'UPRT	Expression of mCherry-tagged MyoF from the UPRT locus	Devarakonda et al., 2023; PMID:37732764
pDHFR-ActinCB-EmeraldGFP	Fluorescent labeling of actin	Periz et al., 2017; PMID: 28322189
pTub-SAG1ΔGPI-mCherry	Fluorescent labeling of Dense Granules	Heaslip et al., 2016; PMID: 27146112
ptub-Grasp55-mCherry	Fluorescent labeling of the Golgi	Pelletier et al., 2002; PMID: 12152082
pTub-FNR-RFP	Fluorescent labeling of the Apicoplast	Vollmer et al., 2001; PMID: 11056177
pU6-5PX1	Targets Cas9 to 5' of exon 1 of the Ku80 locus	O'Shaughnessy et al., 2020; PMID: 32073987
pSAG1::CAS9-U6::sgUPRT	Targets Cas9 to the UPRT locus	Shen et al., 2014; PMID: 24825012; Addgene: 54467

Table S2: List of primers used in this study.

Primer Name	Sequence	Purpose
5'ActinCB-CRISPR-HR-FWD (F1)	actcgttggacacgccgcgtgtaatagagggccggCCCTACCGTCGACGGTATCGATAAG	Amplifies pDHFR-ActinCB-EmFP with homology to repair CRISPR-mediated cut 5' of exon 1 of the Ku80 locus
3'ActinCB-CRISPR-HR-REV (R1)	gatggacgaaactggaagtcgaaagggaaccCCAACGCGTGCGCAGAAAC	Amplifies pDHFR-ActinCB-EmFP with homology to repair CRISPR-mediated cut 5' of exon 1 of the Ku80 locus
MyoF FWD (F2)	GGAGAGCGGAGCAGGCAAGACAGAAA	Differentiates genomic MyoF and second-copy MyoF CDS integrated into the UPRT locus
MyoF REV (R2)	TCGGGGAAGGGAAGTAATAGATGC	Differentiates genomic MyoF and second-copy MyoF CDS integrated into the UPRT locus
5'-UPRT FWD (F3)	TTTTCCTGTTTTCGTCGTCATC	Amplifies pMyoF-mCherry-MyoF with 5' homology to repair CRISPR-mediated cut in UPRT locus
3'-UPRT REV (R3)	TACCACTTCGCTTCCCTGTC	Amplifies pMyoF-mCherry-MyoF with 3' homology to repair CRISPR-mediated cut in UPRT locus

Table S3: List of gene accession numbers associated with this study

Gene Name	ToxoDB Accession Number
Myosin F	TgME49_278870
Tubulin A1	TGME49_316400
Actin	TGME49_209030
UPRT	TGME49_312480
Ku80	TGME49_312510
FNR	TGME49_298990
