# Supplementary material for: Myosin F controls actin organization and dynamics in Toxoplasma gondii
Source: Mol Biol Cell. 2024 Mar 12;35(4):ar57. doi: 10.1091/mbc.E23-12-0510 (PMC11064658; doi:10.1091/mbc.E23-12-0510)
Supplement: Supplementary file 8 [file reviewer_reportE23-12-0510.pdf]

Myosin F controls actin organization and dynamics in Toxoplasma gondii

Jacob Kellermeier and Aoife Heaslip

Corresponding author(s): Aoife Heaslip, University of Connecticut

Review Timeline:

Submission Date:	2023-12-28
Editorial Decision:	2024-01-30
Revision Received:	2024-02-13
Accepted:	2024-02-16

Editor-in-Chief: Matthew Welch

Transaction Report:

(Note: With the exception of the correction of typographical or spelling errors that could be a source of ambiguity, letters and reports are not edited. The original formatting of letters and referee reports may not be reflected in this compilation.)

RE: Manuscript #E23-12-0510
TITLE: "Myosin F controls actin organization and dynamics in Toxoplasma gondii"

Dear Dr. Heaslip:

Thereviewers have expressed support for your work. They have provided valuable feedback, highlighting some minor points that would benefit from your attention.

I trust that addressing these points will be straightforward and not only meet the reviewers' suggestions but will also contribute to enhancing the overall quality of the paper.

Sincerely,
Dominique Soldati
Monitoring Editor
Molecular Biology of the Cell

Dear Dr. Heaslip,

The review of your manuscript, referenced above, is now complete. The Monitoring Editor has decided that your manuscript requires minor revisions before it can be published in Molecular Biology of the Cell, as described in the Monitoring Editor's decision letter above and the reviewer comments (if any) below.

A reminder: Please do not contact the Monitoring Editor directly regarding your manuscript. If you have any questions regarding the review process or the decision, please contact the MBoC Editorial Office (mboc@ascb.org).

When submitting your revision include a rebuttal letter that details, point-by-point, how the Monitoring Editor's and reviewers' comments have been addressed. (The file type for this letter must be "rebuttal letter"; do not include your response to the Monitoring Editor and reviewers in a "cover letter.") Please bear in mind that your rebuttal letter will be published with your paper if it is accepted, unless you have opted out of publishing the review history.

Authors are allowed 15 days to submit a revision. If this time period is inadequate, please contact us immediately at mboc@ascb.org.

In preparing your revised manuscript, please follow the instruction in the Information for Authors (www.molbiolcell.org/info-for-authors). In particular, to prepare for the possible acceptance of your revised manuscript, submit final, publication-quality figures with your revision as described.

If your manuscript contains a Significance Statement, please make sure that it consists of three separate bullet points totaling a maximum of 100 words and addressing each of the following points (see https://www.molbiolcell.org/curation-tools):
First bullet: What is the background context? What gap in knowledge does this study addres?
Second bullet: What are the key findings? What is unique or new about the approach?
Third bullet: Why is this paper significant? How might it influence future research?

To submit the rebuttal letter, revised version, and figures, please use this link (please enable cookies, or cut and paste URL):
Link Not Available

MBoC offers the option to publish your work with immediate open access. Open access can increase the discoverability and usability of your research. If you would like to publish your paper with immediate open access but did not select this option during initial submission, please contact us at mbc@ascb.org..

Authors of Articles and Brief Communications whose manuscripts have returned for minor revision ("revise only") are encouraged to create a short video abstract to accompany their article when it is published. These video abstracts, known as Science Sketches, are up to 2 minutes long and will be published on YouTube and then embedded in the article abstract. Science Sketch Editors on the MBoC Editorial Board will provide guidance as you prepare your video. Information about how to prepare and submit a video abstract is available at www.molbiolcell.org/science-sketches. Please contact mboc@ascb.org if you are interested in creating a Science Sketch.

Thank you for submitting your manuscript to MBoC. Please do not hesitate to contact this office if you have any questions.

Sincerely,

Eric Baker
Journal Production Manager
MBoC Editorial Office
mbc@ascb.org

Reviewer #1 (Remarks to the Author):

Capitalizing on their previous work showing the importance of MyoF and F-actin for the integrity and positioning of post-Golgi compartments (Carmeille et al., 2021, PMID: 33529198), Kellermeier and Heaslip dissect, in the present study, the dynamic localization of MyoF and actin filaments using live microscopy and a collection of organellar fluorescent markers (Golgi, apicoplast, and dense granules) to understand how actin and MyoF coordinate intracellular cargo transport.
By doing so, they convincingly show that both MyoF and actin adopt filament-like structures and that although MyoF depletion causes a fragmentation of the ER and Golgi as well as a defect in apicoplast inheritance, this motor is never observed stably associated with these organelles. Its mode of action remains therefore elusive compared to other cargo-transporting myosins. The particularly interesting point of the study is the observation of changes in the dynamics and organization of the F-actin network in MyoF-depleted parasites. The authors hypothesize that the WD40 domain of MyoF tail would interact with actin (in addition to the motor domain) and therefore would act as a crosslinker. MyoF would thus play a role in filament dispersion during organelle division rather than in carrying cargo directly.

The present study is well conducted with high-quality images, quantifications, and appropriate controls. It is particularly interesting to see the dynamic localization of the proteins of interest without potential artifacts due to the fixation process. For future work, it might be of interest to follow MyoF and ACT in the same cell using two different fluorescent probes to test the filament dispersion model proposed in the discussion.
I don't have major issues that need to be addressed, only a few minor points.

Minor points:
1. The movies are a little fast to appreciate the highlighted biological processes, such as the apicoplast elongation for example.
2. Lines 170 and 289: what is AIP? Unless I am mistaken, this protein is not mentioned in the 2 cited references.
3. Figure 3: The citations of panels B and C are inverted in the figure legend.

Reviewer #2 (Remarks to the Author):

This is a very nicely written and interesting paper on Toxoplasma gondii MyoF. The authors show, using microscopy experiments, that the localization of actin is dependent on the presence of MyoF. My only major concern about the paper is that the authors repeatedly refer to filament-like or filamentous-like structures of both actin and MyoF, and I think that this conclusion cannot be made based on their results, given the resolution of their light microscopy experiments. Actin filaments are about 7 nm thick, myosin filaments about twice that, and even the thickest actin filament bundles should only be less than 150 nm. Looking at the scale bars of the figures (1-5 micrometers), it should not be in any way possible to know whether there are filaments, aggregates, or monomers concentrated in these regions. The authors even refer to the "ends" or "tips" of F-actin bundles (lines 254-270). Even if these actin clusters could be interpreted as filaments or bundles of them, it would not be in any way possible to know where the ends are. I recommend that the authors reword their conclusions accordingly. Already the fact that MyoF affects the localization of actin is interesting in itself. For the future, but likely out of the scope of this revision, it would be interesting to look into this using correlative light and electron microscopy.

The effect of jasplakinolide on the localization of actin is also very interesting. The authors state: "It is unclear why actin localizes to the apical and basal ends of the parasites upon Jas treatment, presumably these actin filament bundles are nucleated by formins 1 and 3 which localize to the apical and basal ends of the parasite, respectively (Tosetti et al., 2019)." I am not sure the change of localization upon jasplakinolide treatment has much to do with what nucleators are present. What other factors could lead to the non-cytosolic distribution of jasplakinolide-stabilized actin? On line 268, they also make comparisons of filament lengths, and this is also not something that can be deduced from these images. At this resolution, the images only show the localization and the length of the area where actin is localized, not filament lengths.

Not being very familiar with the microscopy methods, it is unclear to be, whether the micrographs shown are the only ones imaged and conclusions drawn thus from single observations. I couldn't find this information in the text. It would be nice to know how representative these images are.

I am not sure how much the AlphaFold model of the WD40 domain of MyoF adds to the paper. It is only referred to once in the sense that there is a predicted WD40 domain, but this was already known, and no details are given or conclusions drawn from the predicted atomic model.

In the abstract, the abbreviation MyoF-EmFP is not explained.

Also in the abstract (l. 24), "we find" should be "we found".

The abbreviation ROI (region of interest maybe?) is not explained, as far as I can see.

Throughout the paper, the authors should be careful to use the past tense when describing their observations.

I think the significance statement is not very easily understandable to the general audience and could be made clearer and to better reflect the results.

Reviewer #1 (Remarks to the Author):
Capitalizing on their previous work showing the importance of MyoF and F-actin for the integrity and positioning of post-Golgi compartments (Carmeille et al., 2021, PMID: 33529198), Kellermeier and Heaslip dissect, in the present study, the dynamic localization of MyoF and actin filaments using live microscopy and a collection of organellar fluorescent markers (Golgi, apicoplast, and dense granules) to understand how actin and MyoF coordinate intracellular cargo transport.
By doing so, they convincingly show that both MyoF and actin adopt filament-like structures and that although MyoF depletion causes a fragmentation of the ER and Golgi as well as a defect in apicoplast inheritance, this motor is never observed stably associated with these organelles. Its mode of action remains therefore elusive compared to other cargo-transporting myosins.
The particularly interesting point of the study is the observation of changes in the dynamics and organization of the F-actin network in MyoF-depleted parasites. The authors hypothesize that the WD40 domain of MyoF tail would interact with actin (in addition to the motor domain) and therefore would act as a crosslinker. MyoF would thus play a role in filament dispersion during organelle division rather than in carrying cargo directly.
The present study is well conducted with high-quality images, quantifications, and appropriate controls. It is particularly interesting to see the dynamic localization of the proteins of interest without potential artifacts due to the fixation process. For future work, it might be of interest to follow MyoF and ACT in the same cell using two different fluorescent probes to test the filament dispersion model proposed in the discussion.
I don't have major issues that need to be addressed, only a few minor points.
Minor points:
1. The movies are a little fast to appreciate the highlighted biological processes, such as the apicoplast elongation for example.

The movies have been slowed down by 50% to allow appreciation of the biological processes.

2. Lines 170 and 289: what is AIP? Unless I am mistaken, this protein is not mentioned in the 2 cited references.

The author is correct, AIP was not mentioned in the referenced articles and has been removed as coronin alone suffices as an example of a WD40-containing actin binding protein.

3. Figure 3: The citations of panels B and C are inverted in the figure legend.

This correction has been made.

Reviewer #2 (Remarks to the Author):
This is a very nicely written and interesting paper on Toxoplasma gondii MyoF. The authors show, using microscopy experiments, that the localization of actin is dependent on the presence of MyoF.
My only major concern about the paper is that the authors repeatedly refer to filament-like or filamentous-like structures of both actin and MyoF, and I think that this conclusion cannot be made based on their results, given the resolution of their light microscopy experiments. Actin filaments are about 7 nm thick, myosin filaments about twice that, and even the thickest actin filament bundles should only be less than 150 nm. Looking at the scale bars of the figures (1-5 micrometers), it should not be in any way possible to know whether there are filaments, aggregates, or monomers concentrated in these regions.

We agree with the review on this point, we are unable to distinguish single actin filaments from actin bundles or discern actin filament lengths. To address this valid concern, a caveat statement has been added to the beginning of the results section (line 124) which reads, "Due to the resolution limit of light microscopy, we are unable to determine if these filamentous-like structures are individual filaments or actin bundles, nor are we able to discern actin filament lengths. Thus, any reference to the filament/bundle organization of MyoF and actin are to provide a qualitative description of protein organization."

The authors even refer to the "ends" or "tips" of F-actin bundles (lines 254-270). Even if these actin clusters could be interpreted as filaments or bundles of them, it would not be in any way possible to know where the ends are. I recommend that the authors reword their conclusions accordingly. Already the fact that MyoF affects the localization of actin is interesting in itself. For the future, but likely out of the scope of this revision, it would be interesting to look into this using correlative light and electron microscopy.

This statement has been rewritten to read "A punctum of MyoF was localized at the basal end of these actin structures" (line 273). For consistency, we have also made the following changes in other places in the text. Line 269 now reads "Jas results in the formation of static bundle-like actin structures at the apical and basal ends of the parasite"**. Line 277 reads "**We were intrigued by the observation that MyoF localized adjacent to the bundle-like actin structures after Jas treatment…**"**

The effect of jasplakinolide on the localization of actin is also very interesting. The authors state: "It is unclear why actin localizes to the apical and basal ends of the parasites upon Jas treatment, presumably these actin filament bundles are nucleated by formins 1 and 3 which localize to the apical and basal ends of the parasite, respectively (Tosetti et al., 2019)." I am not sure the change of localization upon jasplakinolide treatment has much to do with what nucleators are present. What other factors could lead to the non-cytosolic distribution of jasplakinolide-stabilized actin?

We have revised this statement to read "It is unclear why actin localizes to the apical and basal ends of the parasites upon Jas treatment but is potentially due to the action of one or more actin-binding proteins.**" (line 276)**

On line 268, they also make comparisons of filament lengths, and this is also not something that can be deduced from these images. At this resolution, the images only show the localization and the length of the area where actin is localized, not filament lengths.

The statement of MyoF regulating actin lengths has been revised to, "However, in the absence of MyoF, F-actin extended along the parasite periphery, indicating that MyoF also influences actin organization in Jas-treated parasites**." (line 281)**

Not being very familiar with the microscopy methods, it is unclear to be, whether the micrographs shown are the only ones imaged and conclusions drawn thus from single observations. I couldn't find this information in the text. It would be nice to know how representative these images are.

The following statement has been added to the methods section "Representative images shown in figure 2 were chosen from 75 vacuoles (Golgi; 4 independent experiments), 114 vacuoles (apicoplast; 6 independent experiments) and 46 vacuoles (dense granules; 3 independent experiments)**." (Line 442). Figure legends include the statement "**Representative images…**"**

I am not sure how much the AlphaFold model of the WD40 domain of MyoF adds to the paper. It is only referred to once in the sense that there is a predicted WD40 domain, but this was already known, and no details are given or conclusions drawn from the predicted atomic model.

The AlphaFold supplemental figure has been removed, and figure references updated accordingly.

In the abstract, the abbreviation MyoF-EmFP is not explained.

Within the abstract, the statement referring to MyoF-EmFP has been expanded to include the full name of the fluorescent protein, "MyoF-EmeraldFP (MyoF-EmFP)**" for clarification.**

Also in the abstract (l. 24), "we find" should be "we found".

Present tense verbs within the abstract have been changed to past tense.

The abbreviation ROI (region of interest maybe?) is not explained, as far as I can see.

The statements mentioning ROI have been expanded to, “region of interest (ROI)**” for clarification of the abbreviation (figure legends 2 and 3)**

Throughout the paper, the authors should be careful to use the past tense when describing their observations.

Throughout the paper, statements involving our observations written in the present tense have been changed to the past tense.

I think the significance statement is not very easily understandable to the general audience and could be made clearer and to better reflect the results.

The significance statement has been rewritten.

RE: Manuscript #E23-12-0510R
TITLE: "Myosin F controls actin organization and dynamics in Toxoplasma gondii"

Dear Dr. Heaslip:

I am pleased to accept your manuscript for publication in Molecular Biology of the Cell.

The authors have appropriately addressed all the points raised by the reviewers

Sincerely,
Dominique Soldati
Monitoring Editor
Molecular Biology of the Cell

Dear Dr. Heaslip:

Congratulations on the acceptance of your manuscript! Thank you for choosing to publish your work in Molecular Biology of the Cell (MBoC).

Within 10 days, an unedited PDF of your manuscript will be published on MBoC in Press, an early release version of the journal. The date your manuscript appears on this site is the official publication date.

Your copyedited and typeset manuscript will be scheduled for publication in the next available issue of MBoC. Our production team will notify you when the page proofs of your paper are ready for your review.

MBoC offers the option to publish your paper with immediate open access. Open access can increase the discoverability and usability of your research. If you would like to publish your paper with immediate open access but did not select this option during initial submission, please contact the MBoC Editorial Office (mbc@ascb.org).

We invite you to submit images related to your accepted manuscript to be considered for the journal cover.
Cover images must contain color, TIF Format
Size image at 7.4" wide x 5.85" deep
RGB and CMYK color (please submit two separate files, one in each format)
300 dpi resolution

Please also send a 1-paragraph description of the image that a general audience could understand. Include with the caption the name and institution of the individual(s) the image should be attributed to. Please provide this description in a Word file. You can send the file via email or by your favorite file transfer service to mboc@ascb.org.

Authors of Articles and Brief Communications are also encouraged to create a short video abstract to accompany their article when it is published. These video abstracts, known as Science Sketches, are up to 2 minutes long and will be published on YouTube and then embedded in the article abstract. Science Sketch Editors on the MBoC Editorial Board will guide you as you prepare your video. Information about how to prepare and submit a video abstract is available at www.molbiolcell.org/science-sketches. Please contact mboc@ascb.org if you are interested in creating a Science Sketch.

We look forward to publishing your paper in MBoC.
Sign up for MBoC's new content alerts here https://tinyurl.com/3bcsyk3e

Sincerely,

Eric Baker
Journal Production Manager
MBoC Editorial Office
mbc@ascb.org
